# Comparative SARS-CoV-2 Omicron BA.5 variant and D614G-Wuhan strain infections in ferrets: insights into attenuation and disease progression during subclinical to mild COVID-19

**DOI:** 10.3389/fvets.2024.1435464

**Published:** 2024-08-15

**Authors:** Sandra Barroso-Arévalo, Lidia Sánchez-Morales, Néstor Porras, Marta Díaz-Frutos, Jose A. Barasona, Julio Isla, Débora López, Christian Gortázar, Lucas Domínguez, Jose M. Sánchez-Vizcaíno

**Affiliations:** ^1^Department of Animal Health, Faculty of Veterinary, Universidad Complutense de Madrid, Madrid, Spain; ^2^VISAVET Health Surveillance Centre, Universidad Complutense de Madrid, Madrid, Spain; ^3^Sabiotec SL, Ciudad Real, Spain; ^4^SaBio, Instituto de Investigación en Recursos Cinegéticos IREC (CSIC-UCLM-JCCM), Ciudad Real, Spain

**Keywords:** SARS-CoV-2, experimental model, pathogenesis, Omicron, ferrets, attenuation

## Abstract

**Introduction:**

As the SARS-CoV-2 virus continues to evolve and new variants emerge, it becomes crucial to understand the comparative pathological and immunological responses elicited by different strains. This study focuses on the original Wuhan strain and the Omicron variant, which have demonstrated significant differences in clinical outcomes and immune responses.

**Methods:**

We employed ferrets as an experimental model to assess the D614G variant (a derivative of the Wuhan strain) and the Omicron BA.5 variant. Each variant was inoculated into separate groups of ferrets to compare disease severity, viral dissemination, and immune responses.

**Results:**

The D614G variant induced more severe disease and greater viral spread than the Omicron variant. Notably, ferrets infected with the D614G variant exhibited a robust neutralizing antibody response, whereas those infected with the Omicron variant failed to produce a detectable neutralizing antibody response. Despite the clearance of the virus from nearly all tissues by 7 days post-infection, an increase in pathological lesions was observed from 14 to 21 days, particularly in those infected with the D614G variant, suggesting a sustained immune response even after viral clearance.

**Discussion:**

These findings underscore the adaptability of SARS-CoV-2 and illuminate how susceptibility and clinical manifestations vary across different strains and species. The results emphasize the necessity of considering both the direct effects of viral infection and the indirect, often prolonged, impacts of the immune response in evaluating the outcomes of SARS-CoV-2 infections.

## 1 Introduction

Severe acute respiratory syndrome coronavirus 2 (SARS-CoV-2) emerged in late 2019, causing the COVID-19 pandemic, a global public health crisis of unprecedented magnitude (1). The virus is believed to have a zoonotic origin (2), with the initially reported cases linked to a seafood market in Wuhan, China, where live animals were also sold. Although the exact animal reservoir remains elusive, genetic analyses have suggested that SARS-CoV-2 likely originated from bats and may have been transmitted to humans through an intermediate host not yet identified (3, 4).

Despite humans being the primary host of the virus, various animal species have been shown to be susceptible to SARS-CoV-2 infection, either through natural or experimental means. Examples include non-human primates (5), farmed mink (6), felines (7), ferrets (*Mustela furo*), dogs (8) and rodents (9), among others. Studying the infection dynamics in these species has been one of the key priorities during the recent pandemic years since there was significant uncertainty regarding the epidemiological role of animals. In an undesirable scenario, animal infections could pose a significant risk to public health. However, research in this area has shed light on virus transmission, host adaptation, and potential spillback events, demonstrating that only certain species, such as farmed mink or wild white-tailed deer (*Odocoileus virginianus*), may act as reservoirs for the virus (10–13). Conversely, common pets (cats, dogs, or ferrets) appear to be sporadically affected by the virus without severe consequences, as demonstrated by numerous studies (14–17). This contrasts with other experimental studied in which higher doses of virus are used triggering effective infections (8, 18, 19). In addition, the potential value of susceptible species as experimental models has also been explored to enhance our understanding of virus pathogenesis and to evaluate vaccines and treatments. Today, the most commonly used experimental models for SARS-CoV-2 investigations are rodents, such as K18-hACE2 mice (20), and Syrian hamsters (21). While rodent models have undoubtedly contributed significantly to our understanding of SARS-CoV-2 pathogenesis, it is essential to recognize the value of exploring naturally susceptible species like ferrets. Ferrets have been widely used as model organisms in respiratory virus research (22), including influenza virus infections (23) and SARS-CoV (24). Ferrets are susceptible to SARS-CoV-2 infection, and the virus replicates efficiently in their respiratory tract. Importantly, infected ferrets and other mustelids such as farmed American mink (*Neovison vison*) can transmit the virus to naive co-housed animals, making them a suitable model for studying virus transmission dynamics (25). Additionally, the clinical manifestations of SARS-CoV-2 infection in ferrets resemble those observed in humans, typically ranging from asymptomatic to mild respiratory symptoms (18, 26). Furthermore, their size allows for more practical collection and analysis of various types of biological samples, aiding in detailed histopathological and virological studies. These characteristics make them an interesting option for evaluating the pathogenesis and evolution of different SARS-CoV-2 variants and for extrapolating the outcomes to human infection.

In humans, SARS-CoV-2 exhibits a distinct preference for the upper and lower respiratory tracts, specifically targeting cell types with high expression of its primary receptor, ACE2. As revealed by single-cell RNA sequencing, ACE2 is highly expressed in type II alveolar epithelial cells (AT2), with an impressive 83% of ACE2-expressing cells in lung tissue being AT2 cells (27). However, ACE2 expression extends beyond the respiratory tract, being well represented in various cell types such as cardiomyocytes, renal proximal convoluted tubule epithelial cells, bladder epithelial cells, and cells in the esophagus, ileum, and Leydig cells (28). Consequently, these tissues and organs may be vulnerable to SARS-CoV-2 infection, providing a basis for the virus’s multi-organotropism. These outcomes may be also applied to other species with similar distribution of ACE2 receptors, such as ferrets (29). Our understanding of SARS-CoV-2 tissue tropism has been primarily established through research on the original strain, the Wuhan variant, or its early derived variant called D614G (30). However, the pandemic landscape has been dynamically shaped by the emergence of new variants, such as the currently dominant Omicron (31), necessitating investigation into the potential shifts in tissue tropism and disease progression. Omicron’s rapid global spread and unique genetic profile, featuring numerous spike glycoprotein mutations-some shared with previous variants, others unique to Omicron-are a testament to its significant adaptive evolution (32). These alterations potentially enhance its capacity for immune evasion, but their impact on viral tissue tropism and pathogenicity remains to be fully elucidated. However, studies have shown that Omicron is associated with significantly lower hospitalization rates than its predecessors, such as the Alpha, Gamma, and Delta variants (33–35). The reasons for the reduced severity of illness caused by Omicron are still being investigated, but some findings suggest that the variant does not replicate as readily in the lower respiratory tract compared to the upper respiratory tract (36, 37). In addition, according to recent research, Omicron displays reduced pathogenicity and enhanced stimulation of anti-inflammatory cytokines. This variant has diminished its capacity to generate inflammatory cytokines while increasing its ability to provoke immunogenic and anti-inflammatory cytokine responses (38).

Despite the existence of published studies using experimental models (39), such as mice (40) or hamsters (41), these models tend to more closely resemble severe cases of the disease. Therefore, it is crucial to investigate the pathogenesis of the Omicron variant in models that mimic light to mild symptoms. To address this need, we have employed the ferret model to examine the clinical progression, excretion patterns, and lesion development as well as viral distribution in animals infected with both the D614G-Wuhan variant and the Omicron BA.5 variant. At the inception of our study, Omicron BA.5 was one of the predominant sublineages of the SARS-CoV-2 virus, causing significant outbreaks and becoming the leading strain in certain regions, such as Spain (42). Consequently, its selection for our research was pertinent and timely, given its relevance in the ongoing global pandemic. Although more recent sublineages like XBB have since emerged and overtaken BA.5 in prevalence (43), the importance of understanding the pathogenicity and host response to Omicron remains undiminished. Each investigation into a specific sublineage contributes to our broader comprehension of the virus’s dynamics and the diverse manifestations of COVID-19. It is crucial to remember that while the virus continues to evolve, the insights we gain from studying each variant and sublineage fortify our collective knowledge base, enhancing our preparedness for future challenges and aiding in the design of effective control strategies and therapeutics.

## 2 Materials and methods

### 2.1 Ethical considerations and animal welfare

The care and procedures involving animals adhered to the principles of good experimental practices as outlined in the Code of Practice for Housing and Care of Animals Used in Scientific Procedures. This code was endorsed by the European Economic Community in 1986 (86/609/EEC, amended by Directive 2003/65/EC) and the Spanish legislation (RD 53/2013). Additionally, the protocol received approval from the Ethics Committee of the Madrid Community (reference PROEX 165.3/22) and the Ethics Committee for Animal Experiments at Complutense University of Madrid (Project License 14/2020). The approved protocol encompassed a comprehensive account of the measures taken to offer environmental enrichment and minimize animal suffering, including humane endpoints and guidelines for euthanasia.

### 2.2 Study design

**Figure 1:**
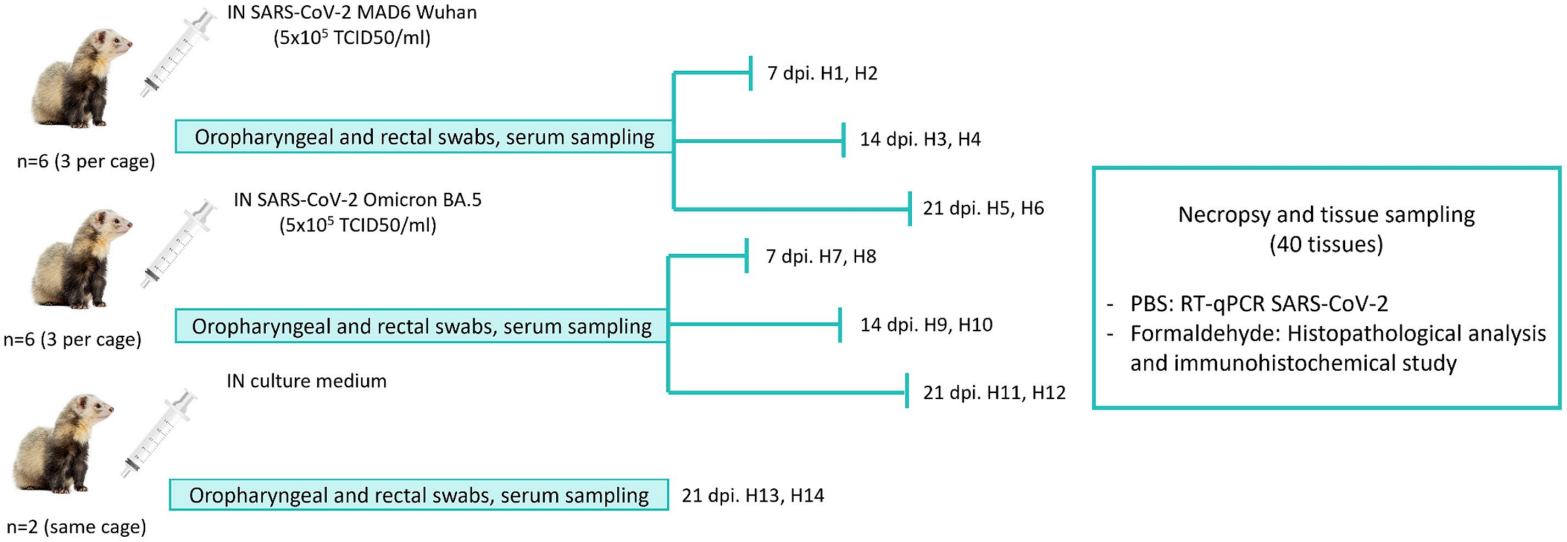
Outline of the experimental design carried out on animals H1–H6 infected with the D614G-Wuhan variant, H7–H12 infected with the Omicron BA.5 variant, and H13 and H14 animals from the control group (IN, intranasal; dpi, day post-infection; TCID50, 50% tissue culture infectious dose).

A total of 14 ferrets (10 females and 4 males) were obtained from private owners in the Castilla la Mancha Community, Madrid, Spain. A clinical examination was performed in Sabiotec,^1^ together with blood extraction for blood count and biochemical parameters, to ensure the good health condition of the animals. Additionally, “INgezim Moquillo IgG” kit (Ingenasa, Madrid, Spain) was employed to confirm the ferrets were not exposed to canine distemper virus. After confirming the animals were in good health condition, three groups were established as follows: D614G-Wuhan (*n* = 6; H1, H2, H3, H4, H5 and H6); Omicron BA.5 (*n* = 6; H7, H8, H9, H10, H11, H12); and Control (*n* = 2; H13, H14). The 12 ferrets from D614G-Wuhan and Omicron BA.5 were arranged in groups of three animals per cage (maintaining animals from each group together) and located in the Biosafety Level 3 (BSL3) area at the VISAVET Surveillance Center (Madrid, Spain). In adherence to animal welfare protocols, ferrets were not housed in microisolator cages. A spatial arrangement was maintained with a minimum of two meters between cages to mitigate aerosol transmission risks. Enhanced ventilation protocols were in place to further reduce potential viral spread. The experimental design is shown in Figure 1. Animals from the Control group were hosted in the BSL2 area of the same center (two animals in the same cage). After 1 week of adaptation, animals were challenged with 1 mL (500 μL per nostril) of the corresponding virus (Wuhan-D614G and Omicron BA.5) intranasally at a final concentration of 1 × 10^5^ TCID/50 under sedation with dexmedetomidine (0.01 mg/kg). Paired oropharyngeal and rectal swabs were collected from each ferret three times per week (2, 4, 7, 9, 11, 14, 16, 18 and 21 dpi) using DeltaSwab^®^ Virus with 3 mL of viral transport media (VTM) (Deltalab S.L., Cataluña, Spain). Sera were also collected before infection and at 4, 7, 9, 14, 16, 18, and 21 dpi (days post-infection).

Animals were euthanized at 7, 14, and 21 dpi using 4 mL of sodium pentobarbital (Dolethal, Vetoquinol Especialidades Veterinarias, S.A., Madrid, Spain) intravenously, and were subjected to a systematic necropsy. Samples from the following tissues were collected in 10% neutral formalin in order to assess histopathological changes and immunohistochemical analysis and in 1 mL of phosphate buffered saline (PBS) to evaluate the presence or absence of SARS-CoV-2 throughout reverse transcription-quantitative PCR (RT-qPCR): brain, cerebellum, spinal cord, bone marrow, cervical lymph node, popliteal lymph node, parotid lymph node, retropharyngeal lymph node, submandibular lymph node, mediastinal lymph node, left tracheobronchial lymph node, mesenteric (jejunum) and mesenteric (colon) lymph nodes, parotid and mandibular salivary glands, nasal turbinates, tonsils (palatine and lingual), bladder, trachea, thymus, spleen, heart, left cranial and caudal lobes, right cranial, middle and caudal lobes, accessory lobe, liver, gallbladder, kidney, adrenal gland, gonads, stomach, duodenum, pancreas, jejunum, colon, and rectum. For RT-qPCR, tissues were homogenized using a vortex and then the RNA was extracted using 200 μL of the homogenized sample according to the methodology described in section 4.4.

### 2.3 Virus and cells

The SARS-CoV-2 MAD6 strain was obtained from a male patient in Madrid (44), Spain, and was kindly provided by Dr. Luis Enjuanes from the National Biotechnology Centre (CNB) at the Higher Council for Scientific Research (CSIC). The sequenced genome of the virus was obtained by a previous study and was found to be identical to the SARS-CoV-2 reference strain (Wuhan-Hu-1, GenBank No.: MN908947), with the exception of a non-coding change at C3037>T and two mutations resulting in amino acid alterations: a change at C14408>T within nsp12 and a A23403>G mutation leading to D614G in the Spike protein (45). This isolate is referred as D614G-Wuhan variant for the current study.

The SARS-CoV-2 Omicron BA.5 variant was obtained from an infected patient on May 24, 2022. The virus was isolated from the nasopharyngeal swab (Ct = 17.89), sequenced (EPI_ISL_15809378), and titrated. To produce SARS-CoV-2 stocks, Calu-3 cells were used, which were kindly provided by Dr. Luis Enjuanes. The cells were cultured in Eagle’s Minimum Essential Medium (EMEM) supplemented with 100 IU/mL penicillin, 100 μg/mL streptomycin, 10% fetal bovine serum (FBS), and incubated at 37°C under 5% CO_2_. The titers of SARS-CoV-2 were measured by a tissue culture infectious dose (TCID50) assay and calculated using the Reed–Muench Method (46, 47). Vero E6 cells from ATTC^®^ (Manassas, Virginia) were used for virus isolation and titration. These cells were cultured in RPMI supplemented with 10% FBS, 100 IU/mL penicillin, and 100 μg/mL streptomycin (growth medium).

### 2.4 RNA extraction and reverse transcription-quantitative PCR

The High Pure Viral Nucleic Acid Kit (Roche, Basel, Switzerland) was used to extract total RNA from oropharyngeal, rectal swabs, and other tissues as per the manufacturer’s instructions. The extracted RNA was stored at −80°C after being suspended in RNase-and DNase-free water. To detect SARS-CoV-2 RNA, an reverse transcription-quantitative PCR (RT-qPCR) protocol was used as described in Barroso-Arévalo et al. (48). The standard curve used for copies/microliter quantification is shown in Supplementary Figure S1. The E and RdRp viral genes were used as reference genes. For each sample, viral load Cq values were normalized against these reference genes. Each qPCR run included at least four points from a standard curve generated using a positive control for SARS-CoV-2 real-time RT-PCR. This positive control is an *in vitro* transcribed RNA derived from strain BetaCoV_Wuhan_WIV04_2019 (EPI_ISL_402124), containing amplification regions of the RdRp and E genes. A master bank was prepared at 2 × 10^6^ copies/μL, stored at −80°C, and a working bank at 2 × 10^4^ copies/μL was prepared to avoid freeze/thaw cycles, with working tubes stored at-20°C for less than 1 week to ensure stability. The efficiency of the qPCR was monitored by the slope of the standard curve, with acceptable efficiency ranging between 95 and 105%. All runs met this criterion, confirming the efficiency and reliability of the qPCR reactions. Each curve point was run in triplicate, and the average Cq value was used for subsequent analysis, with discrepancies investigated and outliers excluded if necessary.

Tissues were weighed and placed in tubes containing ceramic beads and 2 mL of PBS. The samples were homogenized using a vortex mixer at maximum speed for 2 min, as previously validated in our laboratory. This process ensures thorough disruption of the tissue and release of nucleic acids. After homogenization, the samples were centrifuged at 13,000 rpm for 5 min to remove solid debris. The supernatant containing the RNA was then collected for further processing.

In addition, to confirm the virus strain present in the RNA samples, an RT-qPCR protocol developed by Sibai et al. (49), which is specifically tailored to distinguish the Omicron variant from other SARS-CoV-2 strains, was used (results not shown).

Tissues were analyzed by RT-qPCR by homogenization.

### 2.5 Detection of neutralizing antibodies

A surrogate SARS-CoV-2 virus neutralization assay (GenScript, Leiden, Netherlands) was employed for identifying neutralizing antibodies, following the guidelines provided by the manufacturer. Samples presenting a cutoff higher than or equal to 30% were considered positive results indicating the presence of SARS-CoV-2 neutralizing antibodies, and samples lower than 30% were considered negative results. Neutralizing antibodies were measured on blood collection days (0, 4, 7, 9, 9, 14, 16, 18, and 21 dpi). To ensure the accuracy of the results, virus neutralization test was performed on Vero cells using the specific isolate of the challenge virus for each group. For the Omicron BA.5 group, the Omicron BA.5 variant was used, while the D614G-Wuhan virus was employed for the D614G-Wuhan group, according to the methodology previously described in Barroso-Arévalo et al. (19).

### 2.6 Virus isolation

All PCR-positive oropharyngeal swab samples were subjected to virus isolation in Vero-E6 cells. The cells were subsequently added to 12-well culture plates and incubated at 37°C with 5% CO_2_ for 24 h. Following this period, the medium was removed, and cells were inoculated with 200 μL of the original sample found in VTM (oropharyngeal swabs). After 1 h of adsorption at 37°C, 200 μL of infection medium (4% FBS) was added. Positive and negative controls were placed on the culture plates. The cells were maintained at 37°C with 5% CO_2_ while monitoring for CPE and cell death daily. After a 3–5-day period, the cell cultures underwent freezing, thawing, and three passages, involving the inoculation of new Vero E6 cell cultures with the lysates, as previously detailed. The molecular detection of SARS-CoV-2 was carried out using RT-qPCR on the supernatants from each passage to verify the presence or absence of the virus in the cell culture and ascertain virus recovery through the decrease in Ct.

### 2.7 Histopathological study

The tissue samples were fixed in 10% neutral formalin for 48 h, automatically processed (Citadel 2000 Tissue Processor, Thermo Fisher Scientific, Waltham, MA), and embedded in paraffin (HistoStar Embedding Workstation, Thermo Fisher Scientific). Five consecutive sections of 4 μm thickness were obtained for each case using a microtome (FinesseMe+, Thermo Fisher Scientific). One section was stained with hematoxylin-eosin (Gemini AS Automated Slide Stainer, Thermo Fisher Scientific), and the following four sections were placed in positively charged glass slides and used for further immunohistochemical studies. The lesions observed by histopathological evaluation were semi-quantitatively scored as follows: negative (0), mild (1), moderate (2), and severe (3). Histopathological assay was conducted by blind observation by two different pathologists.

### 2.8 Inmunohistochemical study

The paraffin sections placed in positively charged glass slides were deparaffinised in xylene and rehydrated. This step was carried out by the Epredia PT module Deparaffin and Heat Induced Epitope Retrieval (HIER). Endogenous peroxidase was blocked by immersing the samples in a 3% hydrogen peroxide in methanol solution (Panreac Química S.L.U.) for 15 min. Then, the samples were incubated with 2.5% Normal Horse Serum (ImmPRESS^®^ VR Horse AntiRabbit IGG Polymer Kit, Vector Laboratories) for blocking (RTU) for 1 h. Afterwards, the slides were incubated overnight at 4°C with the primary antibody. The SARS-CoV-2 N protein Recombinant Rabbit Polyclonal Antibody (elaborated and kindly provided by the Carlos III Health Institute) and the tumour necrosis factor alpha (TNF-α) Rabbit Polyclonal Antibody (AbD Serotec, Bio-Rad Laboratories) were used. Rabbit Positive and negative controls were included in each batch of slides. For negative controls, the primary antibody was omitted and substituted by tris-buffered saline. After the night, the secondary antibody was added (ImmPRESS^®^ VR Horse AntiRabbit IGG Polymer Kit, Peroxidase; Vector Laboratories) and incubated for 1 h. For the revealing process, peroxidase was used (ImmPACT^®^ NovaRED^®^ Substrate Kit Peroxidase). Afterward, the samples were counter-stained with hematoxylin (Gemini AS Automated Slide Stainer, Thermo Fisher Scientific). SARS-CoV-2 distribution and cellular localization were assessed immunohistochemically, characterized by diffuse brown and granular cytoplasmic staining. The virus presence observed by immunohistochemical evaluation was semi-quantitatively scored as follows: negative (0), mild (1), moderate (2), and severe (3). The immunohistochemical study was conducted by blind observation by two different pathologists.

### 2.9 Statistical analysis

Quantitative qPCR values (copies per microliter) in tissues were analyzed using the SPSS 25 statistic program (IBM, Somar, NY, United States) to evaluate potential differences between groups. The data were organized with the following variables: group (indicating the variant), tissue, viral load (copies per microliter) and day post infection for each day of sacrifice. A one-way ANOVA was conducted separately for days 7, 14, and 21 post-infection to compare the viral loads between the two groups. A univariate general linear model was employed to analyze the viral load across different tissues and between the two groups, assessing both main effects and interactions. Spearman correlation analysis was performed to examine the relationships between tissue type, day post-infection, and viral load.

## 3 Results

### 3.1 Clinical signs

Animals from the D614G-Wuhan group showed clinical signs at some point during the experience, including poor coat appearance (3/6), reactive lymph nodes (1/6), or reduced activity (3/6). In the Omicron group, clinical signs highlighted were poor coat appearance (3/6) and loss of weight (2/6). The total clinical score of each animal and clinical score per day are presented in Supplementary Table S2. Overall, clinical signs in both groups remained relatively mild. The complete score results for each clinical sign are shown in Supplementary Table S1.

### 3.2 Viral loads detected in nasal and rectal swabs using qPCR

**Figure 2:**
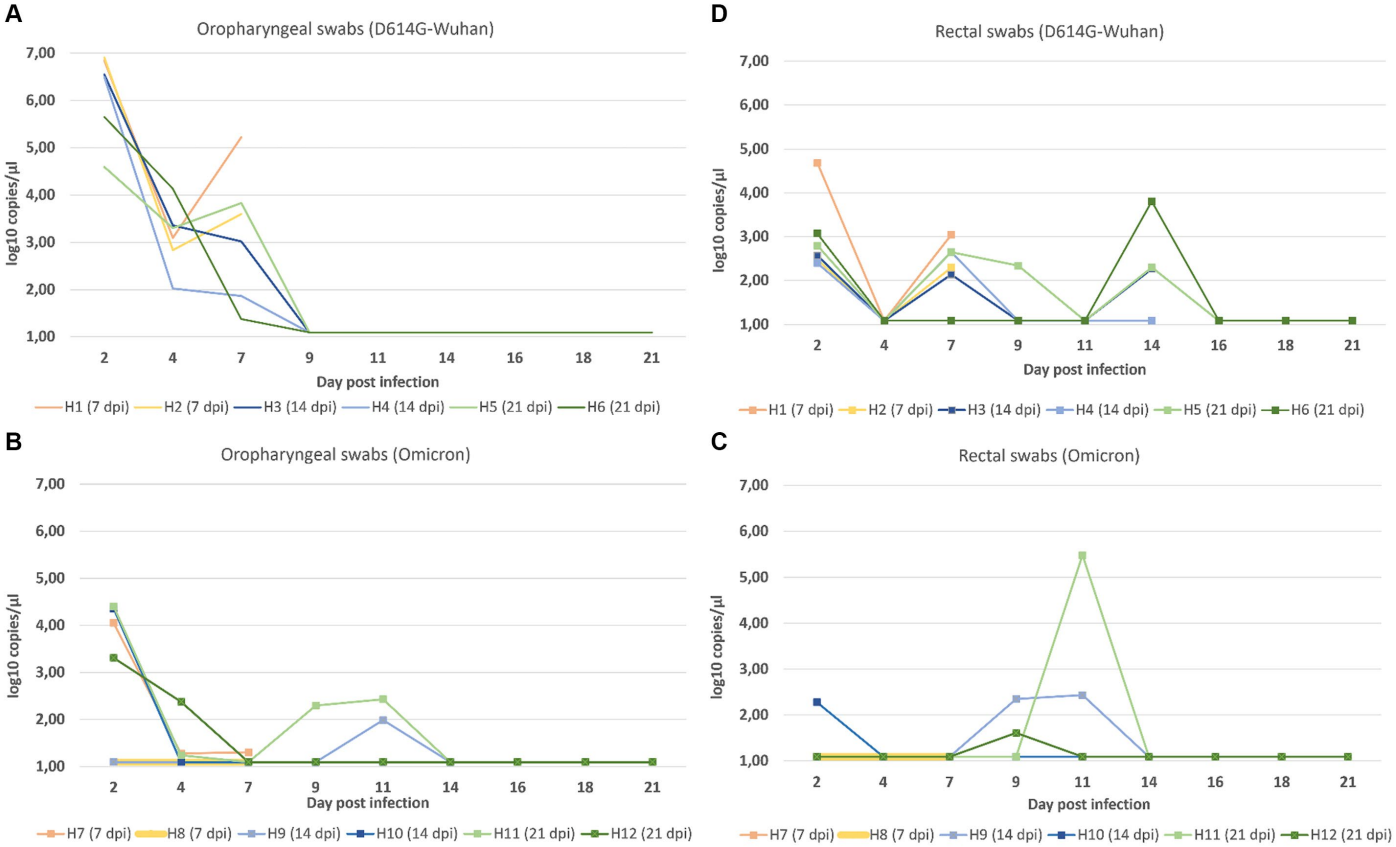
Viral loads based on log copies/μL measured by RT-qPCR for oropharyngeal **(A)** and rectal swabs **(C)** for both the D614G-Wuhan group and the Omicron group (**B**, oropharyngeal swabs; **D**, rectal swabs).

In the D614G-Wuhan group, oropharyngeal swabs displayed exceedingly high viral loads on the 2 dpi, progressively diminishing until the 9 dpi when all animals tested negative. However, in the Omicron group, positive results persisted through to the 14 dpi. Notably, the viral loads obtained from oropharyngeal swabs on day 2 post-infection were markedly higher in the D614G-Wuhan group (Figure 2A) (ranging from 4.59–6.90 log copies/μL) compared to the Omicron group, which showed values between 1.09–4.41 log copies/μL (Figure 2B).

Analyzing the rectal swabs, the viral loads were generally lower in the D614G-Wuhan group again exhibiting higher values than the Omicron group. Yet, interestingly, viral detection in rectal swabs continued until the 14 dpi, a time point at which oropharyngeal swabs were negative. For the animals in the D614G-Wuhan group, the peak viral loads in rectal swabs were observed at 2 dpi and again at 7 dpi (Figure 2C). For the Omicron group, the peaks occurred later, on the 9th and 11 days post-infection (Figure 2D). Control animals were subjected to less frequent sampling compared to those infected with various SARS-CoV-2 strains. Due to their consistent negative results, data from control animals were not included in the graphical representations of our findings.

### 3.3 Viral replication in infected animal tissues

**Figure 3:**
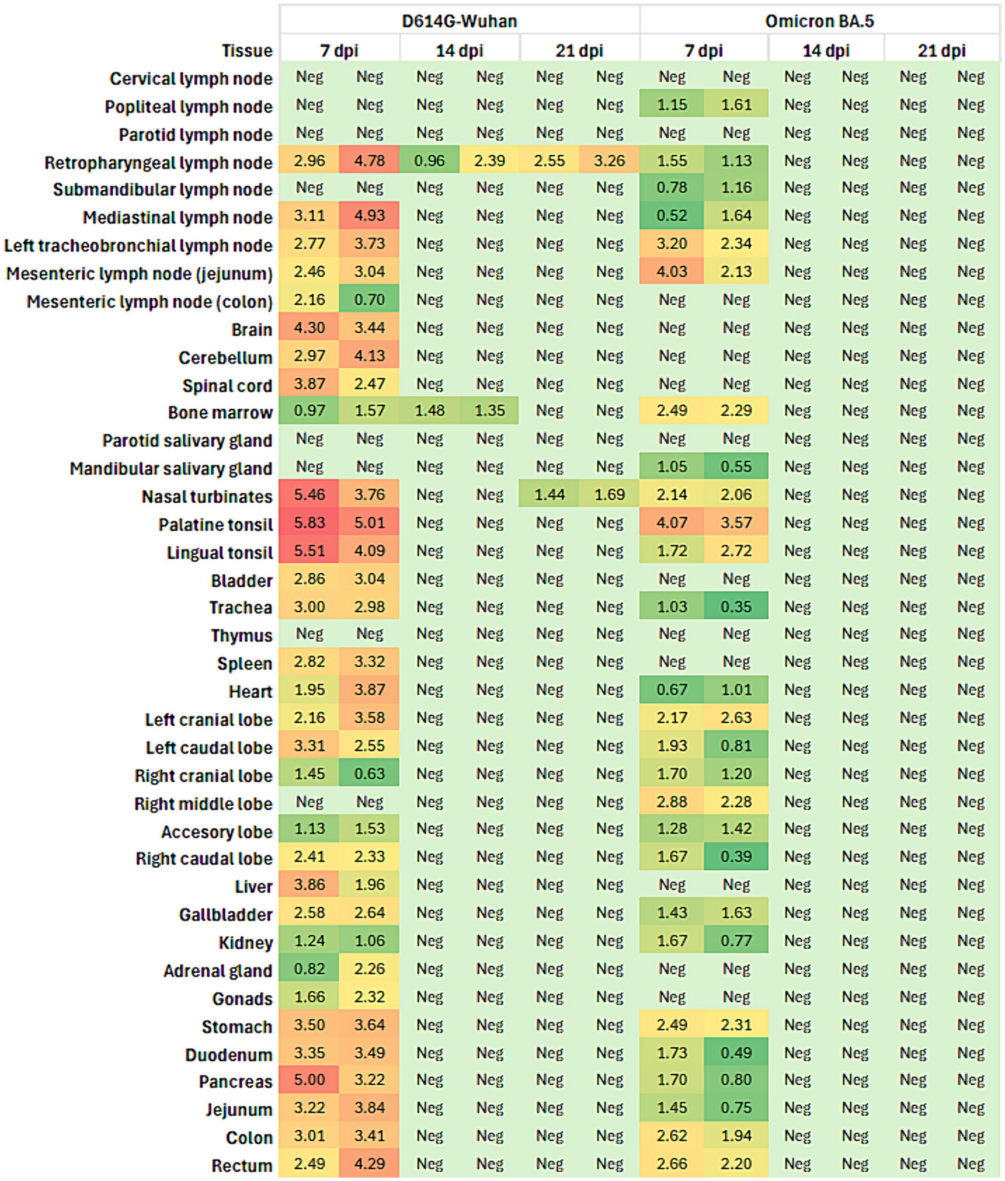
Heatmap illustrating the viral load (in copies/μL) in various tissues from ferrets infected with the D614G-Wuhan variant and the Omicron BA.5 variant of SARS-CoV-2. The data were collected at three different time points post-infection: 7 days post-infection (dpi), 14 dpi, and 21 dpi and represent the average load for each group of two animals sacrificed at each time point. The tissues analyzed include lymph nodes, brain, cerebellum, spinal cord, bone marrow, salivary glands, nasal turbinates, tonsils, bladder, trachea, thymus, spleen, heart, lung lobes, liver, gallbladder, kidney, adrenal gland, gonads, stomach, duodenum, pancreas, jejunum, colon, and rectum. The heatmap uses a color gradient to represent the viral load, with green indicating low viral load and red indicating high viral load. “Neg” indicates tissues where no viral load was detected. The colors in the heatmap correspond to the following ranges of viral load: green: 0–1.5 copies/μL, yellow-green: 1.51–2.5 copies/μL, yellow: 2.51–3.5 copies/μL, orange: 3.51–4.5 copies/μL, red: > 4.5 copies/μL.

Viral loads in 40 tissues of animals from both groups were measured by RT-qPCR. Available viral loads for each animal and tissue are summarized in Supplementary Table S2. For the group inoculated with the D614G-Wuhan variant, the highest detection of viral RNA in tissues was observed in animals culled at 7 days post-infection (dpi). One of the two animals culled at 14 dpi tested negative in all tissues, while the other was positive only for retropharyngeal nodule and bone marrow. In animals sacrificed at 21 dpi, positive results were limited to the retropharyngeal lymph nodes and nasal turbinates. Among the animals culled at 7 dpi, the highest viral loads were detected in the lingual tonsil (5.23 log copies/μL) and palatine tonsil (6.85 log copies/μL) of H1. Moreover, positive results were also found in the retropharyngeal and mediastinal lymph nodes, cerebellum, stomach, and pancreas of both animals (Figure 3). In contrast, for the ferrets infected with the Omicron variant, those sacrificed at 7 dpi displayed positivity for the mesenteric lymph node and nasal turbinates (H7), and for the palatine tonsil and gallbladder (H8) (Figure 3). However, all tissue samples from the animals sacrificed at 14 and 21 dpi tested negative.

### 3.4 Neutralizing antibody detection in sera

**Figure 4:**
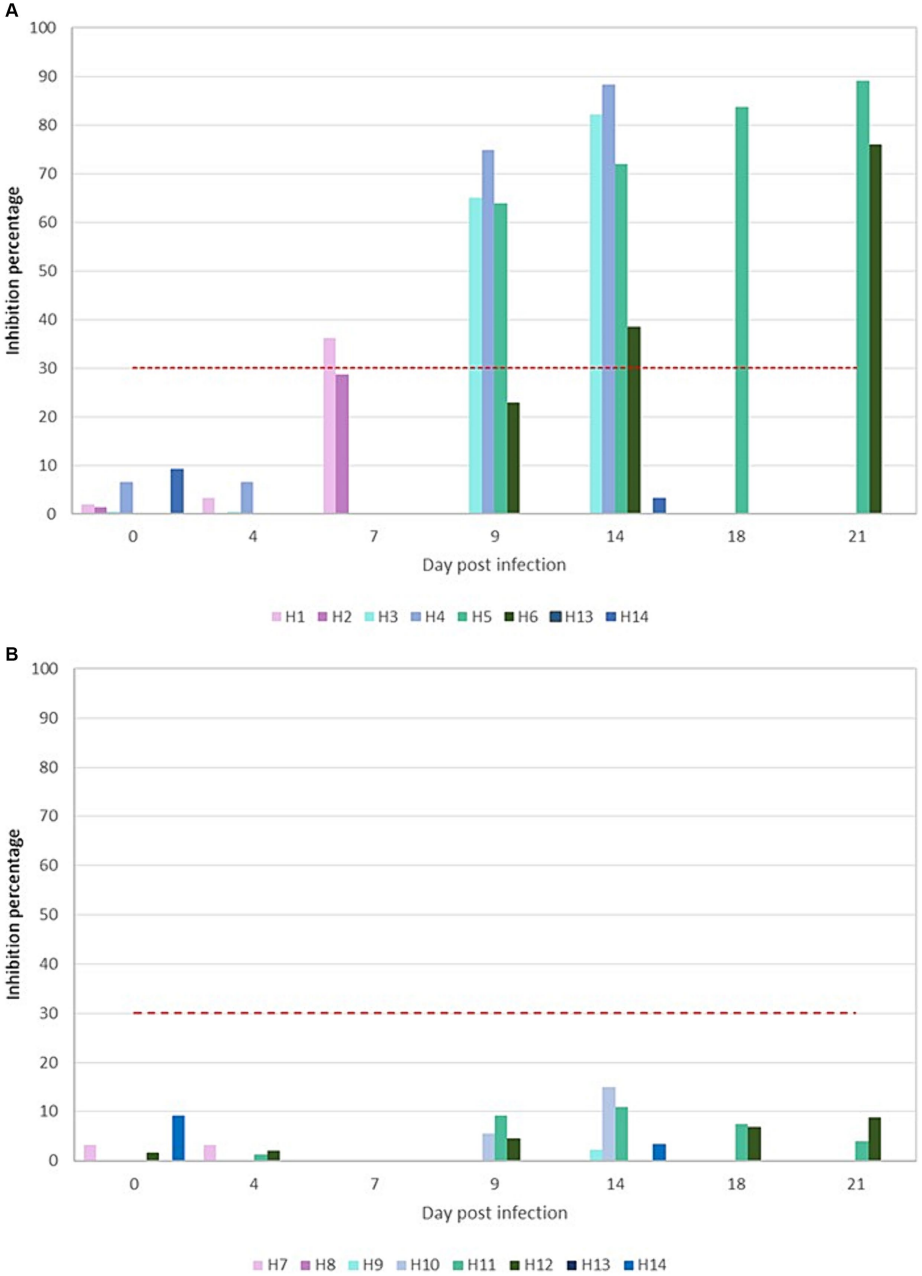
Graphical representation of the percentages of neutralizing antibodies presents in the D614G **(A)** and Omicron BA.5 **(B)** challenged animals. Red discontinuous line represents the cutoff so that higher or equal to 30% results are considered positive.

The assessment of neutralizing antibodies in the serum of the animals revealed distinct outcomes for the two variants. Animals infected with the Omicron variant did not exhibit any detectable antibodies at any point throughout the duration of the experiment (Figure 4B). Conversely, animals infected with the D614G variant began to show neutralizing antibodies from 7 dpi onward, with these levels progressively increasing until they peaked at 89.09% on the 21 dpi (Figure 4A). Similar results were obtained using virus neutralization test with each specific variant.

### 3.5 Virus isolation from oropharyngeal swabs

For the samples taken on day 2 post-infection, all samples from the ferrets in the D614G-Wuhan group tested positive for virus isolation. Additionally, for the Omicron group, 4 out of the 6 ferret samples were found to be positive for viral isolation. By day 4 post-infection, there was a notable shift in the virus isolation results. Every sample from the D614G-Wuhan-infected ferrets remained positive, indicating consistent virus presence. However, in the Omicron group, only one ferret sample out of the six was positive for virus isolation, which may reflect differences in the dynamics of viral presence or isolation efficiency as the infection progresses.

### 3.6 Gross lesions

**Figure 5:**
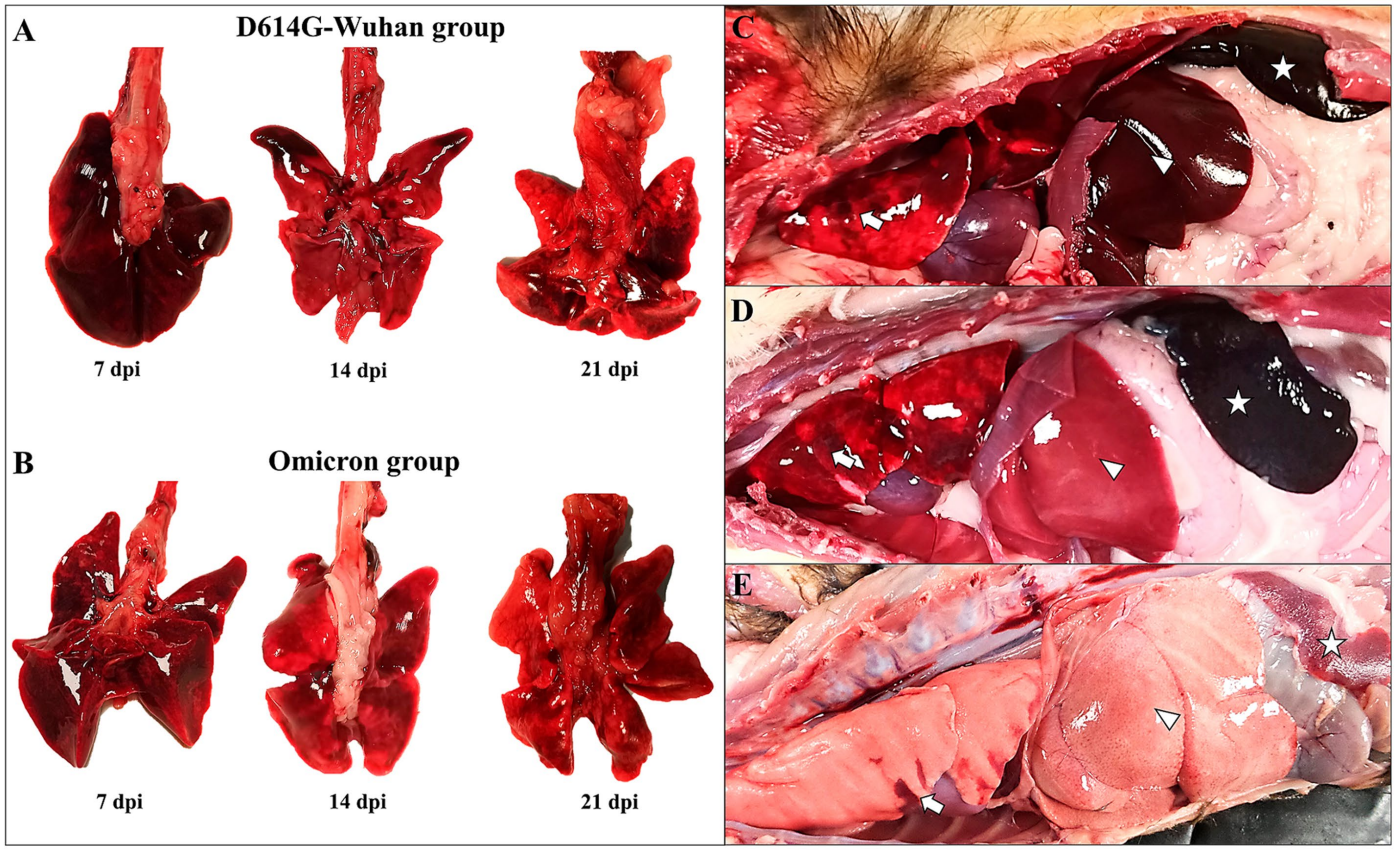
Main necropsy findings in ferrets of both D614G-Wuhan **(A,C)** and Omicron groups **(B,D)**, and control group **(E)**. **(A)** D614G-Wuhan group. Severe lung congestion and edema (7 dpi), interstitial pneumonia with areas of consolidation and hemorrhage in the cranial lobes (14 and 21 dpi) **(B)** Omicron group. Moderate lung congestion (7 dpi); mild areas of consolidation and hemorrhage in the cranial lobe (14 and 21 dpi). **(C,D)** Lung with areas of consolidation and hemorrhage in the cranial lobes (arrow); severe hepatomegaly and white multifocal foci of necrosis **(D)** (arrowhead); severe splenomegaly and hyperemia (star). **(E)** Collapsed lung with small hemorrhagic areas (arrow), severe hepatomegaly and pale liver (arrowhead); exsanguinated spleen (star).

A thorough gross evaluation was conducted for both the D614G-Wuhan and Omicron groups. Two animals from each group were sacrificed at the different time points (7, 14 and 21 dpi). At 7 dpi, the D614G-Wuhan-infected animals exhibited moderate submandibular lymph node lymphadenomegaly, accompanied by minimal petechiae. Their lungs were congestive and edematous, revealing areas of consolidation and hemorrhage, particularly in the cranial and medial lobes (Figure 5A). One out of two animals demonstrated a slight cardiomegaly. The mediastinal and mesenteric lymph nodes appeared intensely hyperemic. Splenomegaly with moderate hyperemia was observed in one animal, while both animals displayed hepatomegaly and intense congestion. The kidneys showed mild medullary congestion with a visible loss of corticomedullary pattern. On 14 and 21 dpi, animals still presented lung congestion and edema, along with images indicative of moderate interstitial pneumonia. There were also continuing areas of consolidation and hemorrhage in the cranial and medial lobes (Figure 5B). All animals exhibited splenomegaly with severe hyperemia. Along with hepatomegaly, marked congestion, and necrotic foci were observed.

In the Omicron group, animals at 7 dpi displayed moderate submandibular lymph node lymphadenomegaly. Their lungs were congestive and edematous, with areas of consolidation and hemorrhage found in the cranial and medial lobes. A mild cardiomegaly was also noted. Both animals exhibited splenomegaly with severe hyperemia, along with hepatomegaly and white multifocal necrotic foci (Figure 5C). At 14 and 21 dpi, animals continued to display lung congestion and edema, with images suggestive of moderate interstitial pneumonia. The persistence of consolidation and hemorrhage in the cranial and medial lobes was observed at 14 dpi. All animals displayed splenomegaly with severe hyperemia, in addition to hepatomegaly and white multifocal necrotic foci (Figures 5D,E).

### 3.7 Histopathological study

Histopathological evaluation was performed in both D614G-Wuhan and Omicron groups. Available histopathological findings reported are summarized in Supplementary Table S3.

**Figure 6:**
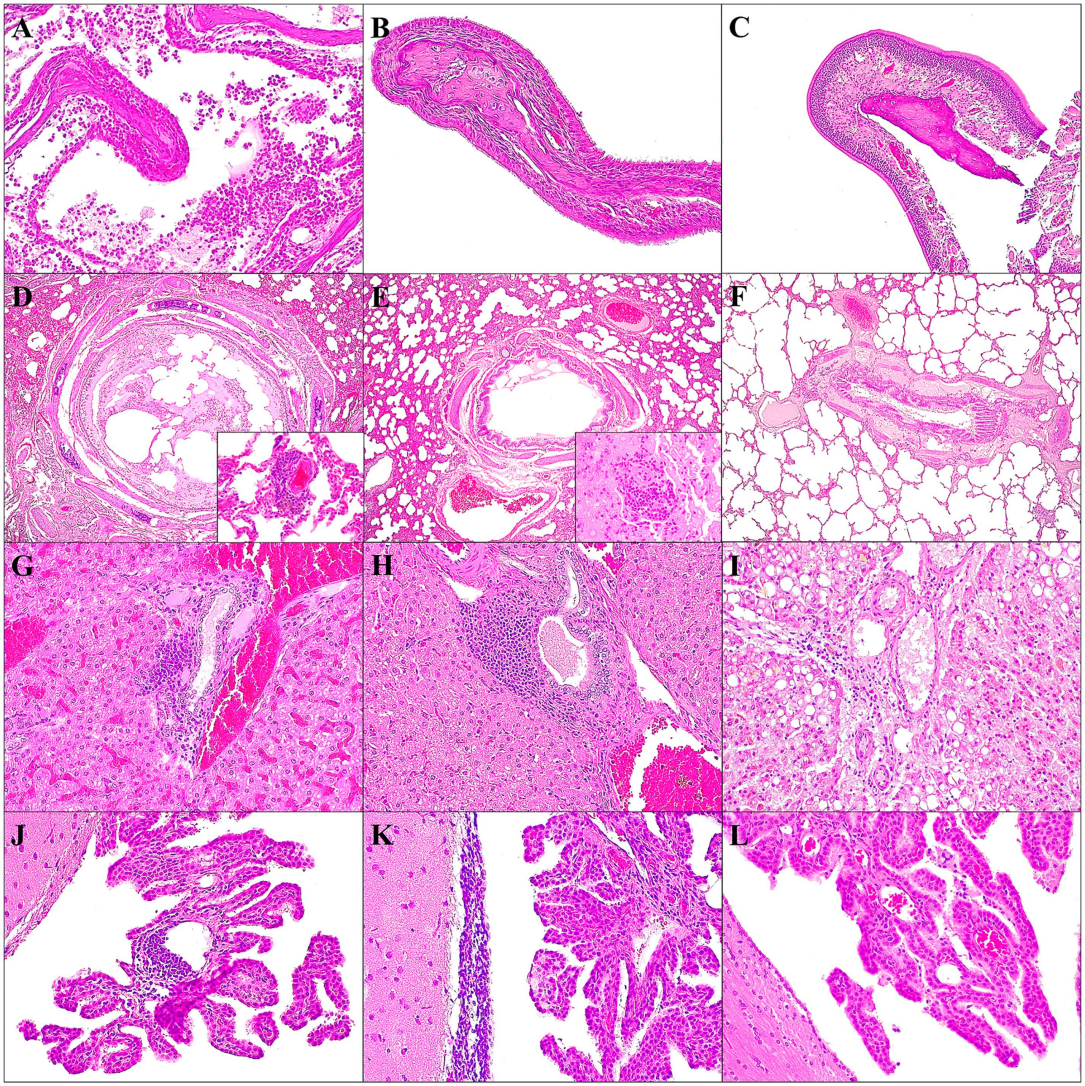
Main histopathological findings observed in ferrets of both D614G-Wuhan group **(A,D,G,J)**, Omicron group **(B,E,H,K)** and control group **(C,F,I,L)**. Lesions observed in nasal turbinates at 7 dpi **(A,B)**; lung at 7 dpi **(D,E)**; liver at 14 dpi **(G,H)**; brain at 21 dpi **(J,K)**. In the control group, histological images of nasal turbinates **(C)**, lung **(F)**, liver **(I)** and brain **(L)** are shown. **(A)** Severe erosive and limphoplasmocytic rhinits with acute suppurative luminal inflammation and mucous exudate; H&E stain, 20×. **(B)** Mild submucosal congestion, with minimal detachment of the epithelial respiratory cells, mainly affecting the apical part, with the removal of the cilia; H&E stain, 20×. **(C)** No epithelial lesions or inflammatory cells were observed in the nasal turbinates; H&E stain, 20×. **(D)** Mild to moderate interstitial pneumonia with congestion; severe bronchus epithelial hyperplasia and detachment; H&E stain, 4×. Inset: perivascular lymphoplasmacytic cuffings; H&E stain, 40×. **(E)** Mild interstitial pneumonia with congestion; mild bronchus epithelial hyperplasia and detachment; H&E stain, 4×. Inset: perivascular lymphoplasmacytic cuffings; H&E stain, 40×. **(F)** No epithelial lesions of the bronchus/ bronchioles and no inflammatory cells were observed in the airways or lung interstitium; H&E stain, 4×. **(G)** Moderate multifocal lymphoplasmacytic periportal hepatitis and congestion; H&E stain, 20×. **(H)** Severe multifocal lymphoplasmacytic periportal hepatitis; H&E stain, 20×. **(I)** Hepatocytes with macrovacuolar degeneration and minimal presence of periportal lymphocytic cells; H&E stain, 20×. **(J)** Moderate focal perivascular lymphocytic inflammation at choroid plexus; H&E stain, 20×. **(K)** Moderate periventricular subependymal lymphocytic inflammation; H&E stain, 20×. **(L)** No inflammatory cells were observed at choroid plexus or periventricular areas; H&E stain, 20×.

In the D614G-Wuhan group, one of the two animals sacrificed at 7 dpi presented intense erosive lesions in the mucosa of the nasal turbinate with acute luminal inflammation composed mainly of neutrophils and mucous exudate. Mild submucosal congestion, edema, and lymphoplasmacytic inflammation were observed, with variable degeneration and detachment of the epithelial respiratory cells, mainly affecting the apical part, with the removal of the cilia (Figure 6A). At 14 dpi, a slight congestion and edema of the submucosa with minimal presence of lymphoplasmacytic cells were observed, in addition to a slight erosion of the apical area of the epithelial cells remained, with a slight decrease in the number of cilia. No significant histopathological findings were observed at 21 dpi. In the trachea, only minimal lymphoplasmacytic tracheitis was observed. In the lung, peribronchial/peribronchiolar lymphoplasmacytic infiltrate was observed, with epithelial hyperplasia in addition to necrosis and epithelial detachment, with a mixture of inflammatory cells and luminal fibrin, with a partial obstruction of the lumen in one of 7 dpi animals (Figure 6C). The other ferret showed a mild to moderate suppurative bronchopneumonia. Both cases manifested multifocal alveolar damage, characterized mainly by hyperplasia of type II pneumocytes, together with moderate alveolar congestion and edema. The alveolar lumen also showed the presence of alveolar macrophages and desquamated type II pneumocytes. They also presented interstitial pneumonia with moderate thickening of the alveolar walls due to mononuclear and neutrophilic inflammatory cells; occasionally an intra-alveolar infiltrate of foamy macrophages was observed. Multifocally, there were perivascular lymphocytic cuffs (Figure 6C). At 14 and 21 dpi, same lesions remained, with an increase in their severity, with frequent obstruction of the bronchi/bronchioles due to mixed inflammation and detached epithelial cells in the lumen. In addition, further collapse of the lung due to the severe diffuse interstitial inflammatory infiltrate was observed, with multifocal foamy macrophage infiltrates and marked perivascular lymphocytic cuffs. The liver at 7 dpi, showed moderate inflammation, lymphoplasmacytic, and periportal/centrolobulillar in both animals. At 14 dpi, they also showed foci of hepatocellular necrosis with mixed inflammatory infiltrate, in addition to microvacuolar degeneration (Figure 6E). After 21 dpi, an increase in the intensity of the necrotic foci and periportal/centrolobular inflammatory infiltrate was observed, showing more organized and aggregated forms. Animals sacrificed at 14 and 21 dpi showed moderate multifocal lymphocytic inflammatory infiltrates at the gallbladder mucosa. Lesions in the kidney were infrequent, observing, in one case sacrificed at 7 dpi, acute tubular necrosis and multifocal suppurative pyelonephritis, with the presence of bacterial colonies in the lumen of the medullary tubules. Both cases at 7 dpi also showed nephrocalcinosis. Only one case (21 dpi) presented moderate multifocal lymphoplasmacytic interstitial nephritis. The main histological finding in the spleen was an intense hyperemia of the red pulp, dilated splenic sinuses with many red blood cells and follicular hyperplasia. The lymph nodes observed in this study showed, in general, without significant differences between the two groups and the dpi, reactive follicular hyperplasia due to an increase in the size of the germinal center, in addition to sinus histiocytosis. This finding was more frequent mainly in retropharyngeal, submandibular, and mediastinal lymph nodes. In the brain, at 7 dpi, an increase in the glial cell population was observed, in addition to a slight multifocal non-suppurative leptomeningitis and periventricular subependymal lymphocytic inflammation. At 14 and 21 dpi, slight to moderate perivascular lymphocyte cuffs were also observed at the neural parenchyma, and moderately in the leptomeninges and choroid plexuses (Figure 6H). No significant lesions were observed in the rest of the organs and tissues studied (see Supplementary Figure S2 for more detailed information).

**Figure 7:**
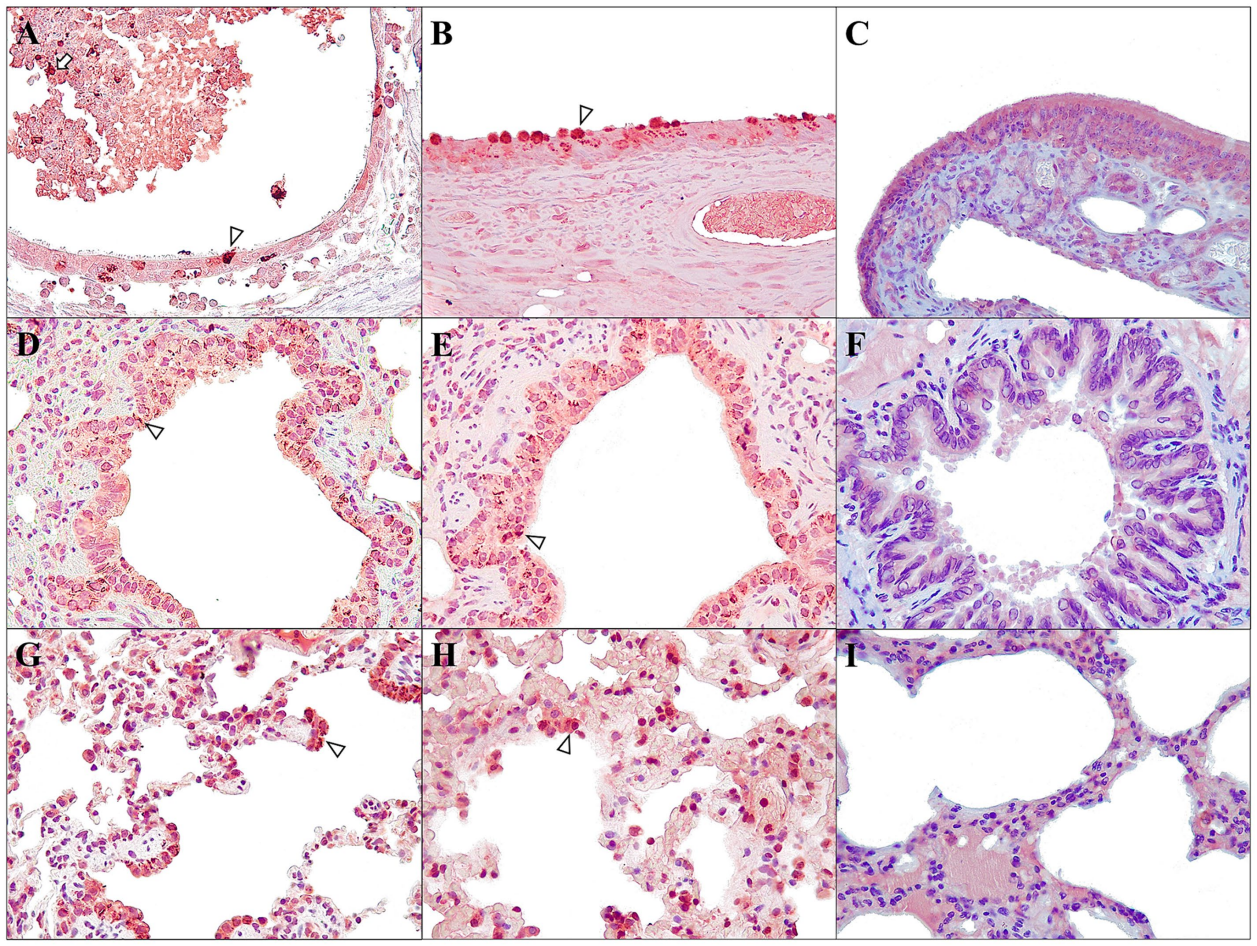
Main immunohistochemical findings in ferrets of both D614G-Wuhan **(A,C,E)** and Omicron groups **(B,D,F)** at 7 dpi, and control group **(C,F,I)**. SARS-CoV-2 distribution and cellular localization analysed in nasal turbinates **(A–C)**, bronchiole **(D–F)**, and lung interstitium **(G–I)**. **(A,B)** Moderate immunoexpression in olfactory epithelial cells (arrowhead) and sloughed epithelium and luminal exudate (arrow); rabbit polyclonal anti-SARS-CoV-2, 40×. **(C)** No immunoexpression was observed in the olfactory epithelial cells; rabbit polyclonal anti-SARS-CoV-2, 40×. **(D,E)** Moderate immunoexpression in bronchiolar epithelium (arrowhead); rabbit polyclonal anti-SARS-CoV-2, 40×. **(F)** No immunoexpression was observed in the bronchiolar epithelial cells; rabbit polyclonal anti-SARS-CoV-2, 40×. **(G,H)** Moderate to intense immunoexpression in the interalveolar cells (macrophages and pneumocytes) (arrowhead); rabbit polyclonal anti-SARS-CoV-2, 40×. **(I)** No immunoexpression was observed in interalveolar cells; rabbit polyclonal anti-SARS-CoV-2, 40×.

In the Omicron group, in 7 dpi animals, mild submucosal congestion were seen in the nasal turbinates (Figure 7B). At 14 dpi, in one of the two cases, moderate acute luminal suppurative inflammation and mucous exudate were observed; in addition to mild submucosal congestion, edema and lymphoplasmacytic inflammation, with slight degeneration and detachment of the epithelial respiratory cells. At 21 dpi, no histopathological findings were observed. In the lung, peribronchial/peribronchiolar lymphoplasmacytic infiltrate was observed, with mild epithelial hyperplasia in addition to necrosis and epithelial detachment, observing partial obstruction at the lumen (Figure 6D). Both cases manifested multifocal alveolar damage, characterized mainly by hyperplasia of type II pneumocytes, together with moderate alveolar congestion and edema. The alveolar lumen also showed the presence of alveolar macrophages and desquamated type II pneumocytes. One case at 7 dpi presented mild multifocal interstitial pneumonia with thickening of the alveolar walls by mononuclear inflammatory cells (Figure 6D). Multifocally, there were perivascular lymphocytic cuffs. At 14 and 21 dpi, the same lesions remained, with an increase in their severity, especially at 14 dpi, observing frequent obstruction of the bronchi/bronchioles due to mixed inflammation, hyperplasic and detached epithelial cells in the lumen. The bronchus associated lymphoid tissue (BALT) presented an intense hyperplasia. In addition, further collapse of the lung due to the severe diffuse interstitial inflammatory infiltrate was observed, with multifocal foamy macrophage infiltrates and marked perivascular lymphocytic cuffs. The liver, at 7 dpi, showed minimal to mild lymphoplasmacytic and periportal/centrolobulillar inflammation. At 14 dpi, the same type of inflammation is found, being more intense (Figure 6G). They also showed foci of hepatocellular necrosis, in addition to microvacuolar degeneration. After 21 dpi, the necrotic foci remained, but only in one case, a mild to moderate lymphoplasmocytic periportal/centrilobular infiltrate was observed. Lesions in the kidney were infrequent, observing nephrocalcinosis, in one case sacrificed at 7 dpi and mild multifocal lymphoplasmacytic interstitial nephritis in one case at 14 dpi. Intense splenic hyperemia was observed in all animals, with dilated splenic sinuses, many red blood cells, and follicular hyperplasia. In both animals at 7 dpi, a high number of megacaryocytes was observed. The lymph nodes observed in this study showed, in general, without significant differences between the two groups and the dpi, reactive follicular hyperplasia due to an increase in the size of the germinal center, in addition to sinus histiocytosis. This finding was more frequent mainly in retropharyngeal, submandibular, and mediastinal lymph nodes. In the brain, an increase in the glial cell population was observed. At 14, they presented slight multifocal non-suppurative leptomeningitis, being more moderate at 21 dpi (Figure 6). No significant lesions were observed in the rest of the organs/tissues studied (see Supplementary Figure S2 for more detailed information).

### 3.8 Inmunohistochemical analysis

Immunohistochemical evaluation (IHC) was performed in both D614G-Wuhan and Omicron groups. Available immunohistochemical findings reported are summarized in Supplementary Table S4.

Positive results were found mainly in animals sacrificed at 7 dpi from both groups. Both groups, at 7 dpi, expressed immunoreaction in the respiratory tissues (Figures 7A,B). In the nasal turbinates, viral antigens were localized primarily within epithelial cells, frequently localized near the basement membrane and in the goblet cells, but also in degenerate and sloughed epithelium and luminal exudate (Figures 7A,B), but no in the olfactory epithelial cells (Figure 7C). IHC labeling of tracheal ciliated epithelium and glands was very rare. In the lungs, moderate immunolabeling of bronchial/bronchiolar epithelium was observed (Figures 7D,F), with prominent expression in necrotic and sloughed cells. Moderate labeling was also observed within intra-alveolar cells compatible with alveolar macrophages and pneumocytes (Figures 7G,I). The liver in the D614G-Wuhan group showed mild immunoexpression mainly in the hepatocytes, but also, rarely, labelling was observed adjacent to inflammatory cells, Kupffer cells, and sinusoids. In the kidney, marked immunolabeling was observed, only in one case of the D614G-Wuhan group (7 dpi) in areas of inflammation and tubular cells. In the spleen, lymph nodes, and bone marrow, minimal immunoexpression in the macrophages was seen. The brain, in the D614G-Wuhan group (7 dpi), presented mild labeling around the neurons and glial cells (microglia and astrocytes), mainly localized at the cerebral cortex. The rest of the tissues studied were negative to IHC (see Supplementary Figure S3 for more detailed information).

**Figure 8:**
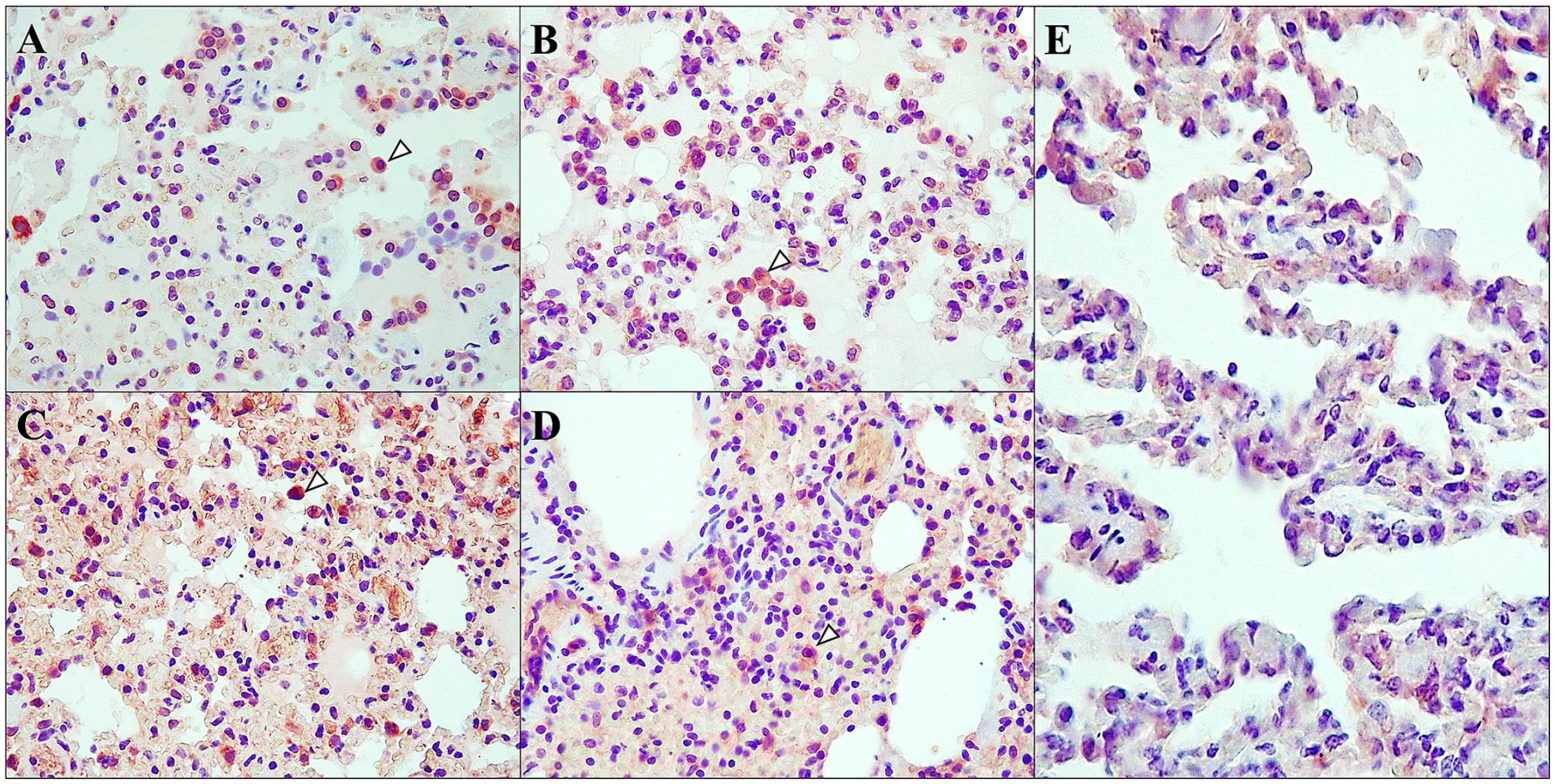
TNF-α distribution and cellular localization. Immunohistochemical analysis of D614G-Wuhan group **(A,C)**, Omicron group **(B,D)** and control group **(E)**; at 7 dpi **(A,B)** and 14 dpi **(C,D)** in lung interstitium. **(A,C)** Moderate to intense immunoexpression in the interalveolar cells (macrophages and pneumocytes) (arrowhead) at 7 and 14 dpi; rabbit polyclonal anti-TNF-α, 40×. **(B)** Intense immunoexpression in the interalveolar cells (macrophages and pneumocytes) (arrowhead) at 7 dpi; rabbit polyclonal anti-TNF-α, 40×. **(D)** Mild immunoexpression in the interalveolar cells (macrophages and pneumocytes) (arrowhead) at 14 dpi; rabbit polyclonal anti-TNF-α, 40×. **(E)** Minimal immunoexpression was observed in interalveolar cells; rabbit polyclonal anti-TNF-α, 40×.

TNF-α immunoexpression was analysed in the lungs in both D614G-Wuhan and Omicron groups. Positive results were found in both groups. The immunoexpression was characterized by a diffuse brown staining, occasionally intensely granular, located in the cytoplasm of certain cells. Positive immunoexpression was found in bronchial/bronchiolar epithelium, with prominent expression in necrotic and sloughed cells. Positive labeling was also observed within intra-alveolar cells compatible with alveolar macrophages and pneumocytes, and, more frequently, in D614G-Wuhan group, the granular immunoexpression located on the endothelium of interstitial capillaries and small to medium-sized blood vessels. Positive immunoexpression was found at different post-infection periods, although more intensely at 7 dpi. The Wuhan group maintained moderate to intense immunoexpression throughout the different post-infection periods, while in the Omicron group, it was prominent at 7 dpi, followed by a lighter immunoexpression at 14 and 21 dpi (see Figure 8 for more detailed information).

### 3.9 Differences in viral load in tissues between groups

The one-way ANOVA for day 7 post-infection indicated a significant difference between the groups [*F*(1, 158) = 25.156, *p* < 0.001], with an effect size (eta squared) of 0.137, suggesting a small to medium effect size. The one-way ANOVA for day 14 post-infection did not show a significant difference between the groups [*F*(1, 158) = 2.011, *p* = 0.158], with a very small effect size (eta squared = 0.013). The one-way ANOVA for day 21 post-infection neither showed significant difference between the groups [*F*(1, 158) = 1.044, *p* = 0.309], with an effect size (eta squared) of 0.007. The univariate general linear model analysis revealed no significant interaction between group and tissue [*F*(39, 400) = 0.911, *p* = 0.627], and no significant main effect for tissue [*F*(39, 400) = 1.125, *p* = 0.284]. The main effect of the group was close to significance [*F*(1, 400) = 2.941, *p* = 0.087]. Spearman correlation analysis indicated no significant correlation between tissue type and day (*r* = 0.000, *p* = 1.000), or tissue type and viral load (*r* = 0.054, *p* = 0.236). A significant negative correlation was found between day post-infection and viral load (*r* = −0.557, *p* < 0.001), suggesting that viral load decreased significantly over time. Supplementary Figure S4 shows comparison of viral loads in all positive tissues between groups.

## 4 Discussion

Our investigation provides novel insights into the comparative outcomes of infections caused by the SARS-CoV-2 D614G-Wuhan strain and the Omicron BA.5 variant within a ferret model, which may represent a subclinical to mild course of infection. We observed marked disparities between both strains in clinical signs, viral replication, antibody responses, and the observation of gross lesions and histopathological alterations. Notably, variations were also evident in the immunohistochemistry findings, evidencing a different viral distribution. The D614G-Wuhan-infected ferrets displayed more evident disease progression and more severe histological lesions than the Omicron-infected ferrets, accompanied by an increase in viral antigens in the respiratory tissues. These observations may contribute to understanding how variations in infection outcomes could potentially manifest between these strains in humans, particularly among individuals presenting asymptomatic infections or extremely mild symptoms. Given the susceptibility of ferrets to SARS-CoV-2 and the resemblance in the number of ACE2 receptors in the respiratory tract to those in humans, this animal model provides useful opportunities for studying SARS-CoV-2, as previously reported (18).

The clinical signs presented in both the D614G-Wuhan and Omicron groups remained mild overall. However, ferrets infected with the D614G-Wuhan strain displayed more intense symptoms, such as reactive lymph nodes and reduced activity, as compared to those infected with the Omicron variant. In addition to the different clinical presentations, a distinct pattern was also observed in viral replication in the oropharyngeal and rectal swabs. Animals infected with the D614G-Wuhan strain exhibited significantly higher viral loads early on at 2 dpi, which decreased until all animals were negative by day 9. On the other hand, ferrets infected with the Omicron variant demonstrated a more intermittent shedding pattern, with positive results extending to 14 dpi but with lower viral loads. Barut et al. (50) observed that the Omicron-BA.1 phenotype failed to replicate effectively in ferrets, indicating a possible lower virulence compared to other variants. This aligns with our findings of reduced clinical severity in Omicron-infected ferrets and provides a broader context for understanding variant-specific pathogenesis. However, Omicron BA.5 has been observed to trigger slightly higher pathogenicity in other animal model (hamster), which may explain the presence of infection in our study. These findings are also in alignment with observations from other experimental models, specifically rodents. In these studies in hamsters and mice, conducted with the Omicron sublineages BA.2 (51), BA.4, and BA.5 (52) the authors observed milder pathogenicity of the Omicron lineages compared to the B.1.617.2 (Delta) variant. These rodent models exhibited efficient viral replication in nasal turbinates but significantly less replication in lung tissues, like what we observed in ferrets infected with the Omicron BA.5 variant. The observed differences in clinical symptoms and viral shedding patterns suggest the possibility of variant-specific adaptations that might have implications for disease progression, transmission dynamics, and infection control strategies. The Omicron variant has demonstrated significant genetic divergence from earlier SARS-CoV-2 strains, which may contribute to its unique pathogenic profile (53). The mutations present in the spike protein of Omicron likely enhance its ability to evade the host immune response and alter its tissue tropism. Previous studies have indicated that Omicron replicates less efficiently in the lower respiratory tract while maintaining high viral loads in the upper respiratory tract, leading to milder clinical symptoms (54). These variant-specific adaptations highlight the importance of continuous surveillance and research to understand the evolving dynamics of SARS-CoV-2.

In this sense, ferrets infected with the D614G-Wuhan strain showed wide viral dissemination, most notably in the nasal turbinates and lungs, but also in the liver, kidney, and brain. This viral distribution correlates with the severe inflammatory lesions observed in these tissues. These observations of the D614G-Wuhan strain’s extensive viral distribution are consistent with Gough et al.’s findings of viral RNA in multiple tissues (55). This highlights the potential for significant viral dissemination beyond the respiratory tract, contrasting with the localized infection seen in Omicron variants. Immunohistochemical analysis reinforced these findings, as we observed a broader presence of viral antigens in the D614G-Wuhan-infected animal tissues. In contrast, ferrets infected with the Omicron variant exhibited a more localized infection, mainly restricted to the upper respiratory tract. Surprisingly, despite the limited viral spread, these animals also presented moderate inflammatory lesions in the liver and, to a lesser extent, in the brain. Nevertheless, these tissues exhibited scarce or absent viral antigens, highlighting the Omicron variant’s limited replicative potential. While it is possible that some of these extra-respiratory lesions may be background lesions, as previous studies suggested (18, 56), reports in SARS-CoV-2 infected mink also showed this type of similar organ involvement in inflammatory lesions (5, 57). These outcomes highlight the virus’s affinity for tissues expressing high levels of the ACE2 receptor, its entry point into host cells (58). Although primarily confined to the upper respiratory tract in our ferret model, which aligns with observations from rodent studies and might account for Omicron’s milder pathogenicity and efficient transmission, our data also indicate reduced yet detectable viral replication in the pulmonary tissues and other organs in fewer animals and at lower viral loads compared to the D614G-Wuhan strain (59). The observed variations in clinical presentations and patterns of viral shedding point to potential differences in how the variants behave, which could inform our understanding of their impact on disease transmission, progression, and the effectiveness of control measures. In the same way, these differences suggest that some factors such as species, viral strain, age, dose, route of inoculation, clinical history, and immune status should be taken into consideration because they could substantially influence disease pathology and viral dissemination (60, 61).

The statistical analyses indicated a significant difference in viral load between the groups on day 7 post-infection. The one-way ANOVA revealed a statistically significant difference [*F*(1, 158) = 25.156, *p* < 0.001], with an effect size (eta squared) of 0.137. This suggests that the ferrets infected with the original variant had a significantly different viral load compared to those infected with the Omicron variant at this early time point. However, this significant difference was not observed on days 14 and 21. The ANOVA results for day 14 [*F*(1, 158) = 2.011, *p* = 0.158] and day 21 [*F*(1, 158) = 1.044, *p* = 0.309] indicated no significant differences, with very small effect sizes (eta squared = 0.013 and 0.007, respectively). This suggests that the initial difference in viral load between the groups diminished over time, leading to comparable levels of viral presence by the second-and third-weeks post-infection. Our data also indicated that despite nearly all tissue clearing the virus by 7 dpi, we observed an increase in pathological findings between 14 and 21 dpi, especially in the D614G-Wuhan group. The increase in these histopathological lesions at 14–21 dpi within the D614G-Wuhan group suggests the presence of a delayed, yet sustained, immune response that continues even after viral clearance from most tissues. The exacerbated immune response, characterized by numerous and significant histopathological lesions, aligns with the noted “cytokine storm” often observed in severe cases of infections caused by the original SARS-CoV-2 virus, where an overactive immune response can result in extensive tissue damage (62–64). This insight is consistent with our immunohistochemical findings, wherein we observed intense TNF-α immunoexpression, one of the key mediators of inflammation, in the lung, remaining elevated primarily in the Wuhan group across different post-infection periods. This observation aligns with findings from some previous studies in ferrets (60, 61). This evidence could suggest that an overactive and potentially pathogenic immune response, rather than the direct damage caused by the virus, could be a significant contributor to the development of the histopathological lesions seen in the D614G-Wuhan group during the study. In light of findings from long COVID research, it can be hypothesized that this sustained immune response is reminiscent of the lingering immune perturbations seen in long COVID, where the immune system continues to cause tissue damage even in the absence of an active virus (65). Recent studies have hinted at the possibility that different SARS-CoV-2 variants might influence the risk of developing long-term symptoms. For instance, while a Swedish study observed a heightened risk of long COVID with infections from the early pandemic isolate, Alpha, and Delta variants compared to Omicron (66), a Norwegian counterpart found Delta and Omicron to have similar rates of long COVID symptoms in adults (67). Yet, a comprehensive review found no significant strain-based differences in long COVID occurrences (68). Interestingly, an Italian observational study hinted at variant-dependent differences in long COVID symptoms. Factors like age, gender, and certain chronic conditions have also been identified as potential risk determinants for long COVID (69). Hence, our findings underscore the importance of considering both the direct effects of viral infection and the indirect, often longer-term, effects of immune response when evaluating SARS-CoV-2 infection outcomes. Furthermore, these observations emphasize the need for therapeutic strategies that address not just viral replication but also manage the potential immunopathological consequences of the infection, highlighting the importance of long-term follow-up and comprehensive management strategies to fully address the ongoing health impacts triggered by the SARS-CoV-2 infection.

These findings have significant implications for understanding the temporal dynamics of COVID-19 infection in ferrets and potentially other similar models. The early significant difference in viral load suggests a possible heightened initial immune response or viral replication rate in one of the groups, which equalizes over time. The lack of significant differences at later stages highlights the importance of longitudinal studies in capturing the full trajectory of viral infections. Future research should focus on expanding the sample size and exploring other physiological and immunological parameters that may explain the initial differences observed. Additionally, further studies could investigate the specific tissue responses and their role in the overall viral clearance process.

Our study also observed interesting differences in the humoral immune response between the D614G-Wuhan and Omicron groups. Animals infected with the D614G-Wuhan strain elicited a strong neutralizing antibody response, which commenced around 7–9 days post-infection. In contrast, the Omicron-infected ferrets did not generate a neutralizing antibody response. These results seem to agree with previous studies (70), suggesting that the localized infection confined to the upper respiratory tract is likely due to the lower replication rate of the virus and its reduced spread. The possibility exists that an innate immune response in the nasal cavity may play a role in modulating the Omicron infection, potentially influencing the activation of B-cells and subsequent antibody production (71). As reflected by recent studies (72), innate immune effectors such as macrophages, dendritic cells, and natural killer cells play a crucial role in the early defense against SARS-CoV-2, potentially contributing to a more controlled infection in the upper respiratory tract. This aligns with findings that effective innate immune responses, including the production of interferons and the activity of natural killer cells, can limit the severity of the disease by restricting viral replication and spread (73). However, this should be interpreted cautiously, and more detailed studies are required to confirm such a relationship. Such a scenario is consistent with the observations made in other species, including rodents (74) and humans, where Omicron infections have frequently demonstrated considerable restriction, occasionally failing to induce a systemic response. While this hypothesis holds plausibility, further research would need to be done to fully understand the underlying immune responses. Specific immune profiling, such as lymphocyte counts, cytokine responses, or quantification of specific immune cells at the local site of infection, may offer enhanced clarity in this regard.

This lower susceptibility to the Omicron variant compared to the original wild-type virus has also been evidenced in other species, such as cats and dogs. Interestingly, a recent study involving cats and dogs from COVID-19 households highlighted that these pets appeared to be less receptive to the Omicron variant compared to earlier variants, including Delta (75). Meanwhile, another investigation into beagle dogs found that these animals were indeed susceptible to both the Delta and Omicron variants of SARS-CoV-2, and they could even serve as transmission vectors for these variants to other dogs, this occurring without any observable clinical signs (76). In contrast, an experimental study with minks involving the Omicron variant (57) reported observable clinical signs, severe histopathological lesions, and high viral loads in swab samples. The observed susceptibility in minks might be due to their being the experimental model that most accurately replicates both the clinical signs and lesions seen in humans (77). In addition, our findings have implications for the monitoring of SARS-CoV-2 in wildlife (78), since animals contacting Omicron (as opposed to D614G-Wuhan and possibly other variants) will less likely present serum antibodies and RNA detection may be restricted to samples from the upper respiratory tract, while lower respiratory tract samples may test negative (79).

In summary, the present experimental study in ferrets, alongside both prior and concurrent studies, provides compelling evidence for the distinct clinical and immunological responses elicited by the D614G-Wuhan strain and the Omicron variant, thereby expanding our understanding of SARS-CoV-2 pathogenesis and immunity. These cumulative findings underscore the complexity and adaptability of SARS-CoV-2, highlighting the varying susceptibility and clinical manifestations in different species. Our study highlights the potential benefits of continuous surveillance and research across diverse animal models to comprehend variant-specific adaptations, which could have far-reaching implications for disease progression, transmission dynamics, and the development of effective monitoring and infection control strategies. However, this study has limitations regarding the number of animals used. While our study was designed with a commitment to the ethical principle of the Three Rs—Replace, Reduce, Refine—resulting in a lower number of animals utilized, we acknowledge this also constrains the breadth of our findings and may limit the statistical power of the results. Further studies could build upon our preliminary insights with a larger cohort to enhance the robustness of the data. Future research should strive to examine the potential role of the immune system in causing long-term damage post-infection, as seen in the “long COVID” phenomenon, and design therapies that address both the direct and indirect effects of the SARS-CoV-2 infection.

## Data availability statement

The original contributions presented in the study are included in the article/Supplementary material, further inquiries can be directed to the corresponding author/s.

## Ethics statement

The animal study was approved by Ethics Committee of the Madrid Community (reference PROEX 165.3/22). The study was conducted in accordance with the local legislation and institutional requirements.

## Author contributions

SB-A: Conceptualization, Data curation, Formal analysis, Investigation, Methodology, Resources, Software, Supervision, Validation, Visualization, Writing – original draft, Writing – review & editing. LS-M: Conceptualization, Data curation, Formal analysis, Investigation, Methodology, Software, Visualization, Writing – original draft, Writing – review & editing. NP: Investigation, Methodology, Validation, Visualization, Writing – original draft, Writing – review & editing. MD-F: Conceptualization, Investigation, Methodology, Visualization, Writing – original draft, Writing – review & editing. JB: Conceptualization, Data curation, Methodology, Supervision, Validation, Writing – review & editing. JI: Methodology, Resources, Writing – review & editing. DL: Methodology, Writing – review & editing. CG: Funding acquisition, Resources, Writing – review & editing. LD: Conceptualization, Funding acquisition, Resources, Supervision, Writing – review & editing. JS-V: Conceptualization, Funding acquisition, Supervision, Writing – review & editing.

## Footnotes

^1^ https://sabiotec.es/

## Supplementary material

SUPPLEMENTARY FIGURE S1 Standard curve used for quantitative RT-qPCR. The standard curve illustrates the direct correlation between the log10 viral RNA concentration (copies/μL) and the RT-qPCR cycle threshold (Ct) values. Analyzed in triplicate, the three lines correspond to the three RT-qPCR targets (green for Sarbeco, orange for IP2, blue for IP4). Each line demonstrates a strong correlation (*R*^2^ values) indicative of the assay’s reliability. The slope of each curve informs on the RT-qPCR reaction efficiency for its respective target.

SUPPLEMENTARY FIGURE S2 Occasional histopathological findings observed in nasal turbinates **(A)**; lung **(B–E)** and kidney at 7 dpi **(G)**. **(A)** Severe erosive and limphoplasmocytic rhinits with removal of the cilia (arrow), and severe luminal neutrophil infiltration (arrowhead). **(B)** Bronchiolar epithelium with hyperplasia and epithelial detachment and pycnosis; the lumen was partially obstructed by an amorphous eosinophilic material, observing a slight deposit of erythrocytes, cellular debris and fibrin (arrow). **(C)** Moderate suppurative infiltration in the bronchiolar lumen (arrowhead); severe bronchus epithelial hyperplasia and detachment. Inset: perivascular lymphoplasmocytic cuffings. **(D)** Mild interstitial pneumonia with scarce number of neutrophils and moderate congestion. **(E)** Moderate intra-alveolar infiltrate of foamy macrophages (arrowhead). **(F)** Acute tubular necrosis and multifocal suppurative pyelonephritis (arrowhead), with the presence of bacterial colonies in the lumen of the medullary tubules (arrow).

SUPPLEMENTARY FIGURE S3 SARS-CoV-2 distribution and cellular localization. Occasional immunohistochemical findings observed in liver **(A)**, kidney **(B)**, bone marrow **(C)** and brain **(D)** at 7 dpi. **(A)** Mild immunoexpression in the hepatocytes adjacent to periportal inflammation. **(B)** Intense immunolabeling adjacent to focal renal pelvis inflammation. Inset (left): immunolabeling in the basement membrane of renal tubules. Inset (right): immunolabeling in the cytoplasm of the inflammatory cells. **(C)** Mild immunoexpression in the cytoplasm of hematopoietic cells. **(D)** Mild immunoexpression in the cytoplasm of neurons and glial cells, mainly in the microglia (arrowhead).

SUPPLEMENTARY FIGURE S4 Comparison of viral loads (copies per microliter) in positive tissues from ferrets infected with D614G-Wuhan (blue bars) and Omicron BA.5 (red bars) variants of SARS-CoV-2.

SUPPLEMENTARY TABLE S1 Complete clinical score for each clinical sign for the animals sacrificed on 7 dpi (D614G-Wuhan group: H1, H2; Omicron group: H7, H8), 14 dpi (D614G-Wuhan group: H3, H4; Omicron group: H9, H10) and 21 dpi (D614G-Wuhan group: H5, H6; Omicron group: H11, H12). Each clinical sign is evaluated from 0 to 2.

SUPPLEMENTARY TABLE S2 Viral loads measured in copies/μl from all the 40 tissues analyzed by RT-qPCR for each animal both in D614G-Wuhan group and Omicron group.

SUPPLEMENTARY TABLE S3 Histopathological scoring in tissues of SARS-CoV-2 infected ferrets.

SUPPLEMENTARY TABLE S4 Immunohistochemical scoring to determine the presence of virus in SARS-CoV-2 infected ferret tissues.
