# Supplementary figures and images for: Comparative SARS-CoV-2 Omicron BA.5 variant and D614G-Wuhan strain infections in ferrets: insights into attenuation and disease progression during subclinical to mild COVID-19
Source: Front Vet Sci. 2024 Aug 15;11:1435464. doi: 10.3389/fvets.2024.1435464 (PMC11358085; doi:10.3389/fvets.2024.1435464)

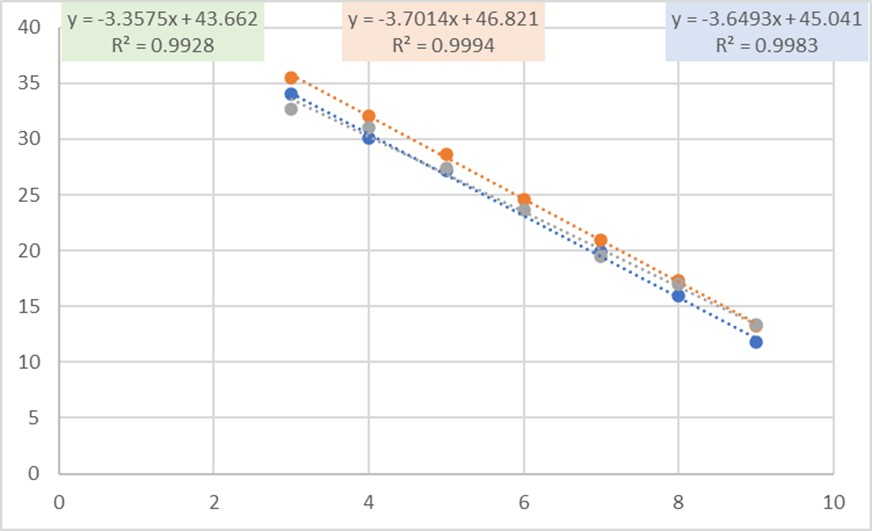

Supplement: SUPPLEMENTARY FIGURE S1 — Standard curve used for quantitative RT-qPCR. The standard curve illustrates the direct correlation between the log10 viral RNA concentration (copies/μL) and the RT-qPCR cycle threshold (Ct) values. Analyzed in triplicate, the three lines correspond to the three RT-qPCR targets (green for Sarbeco, orange for IP2, blue for IP4). Each line demonstrates a strong correlation (R2 values) indicative of the assay’s reliability. The slope of each curve informs on the RT-qPCR reaction efficiency for its respective target. [file Image_1.TIF]

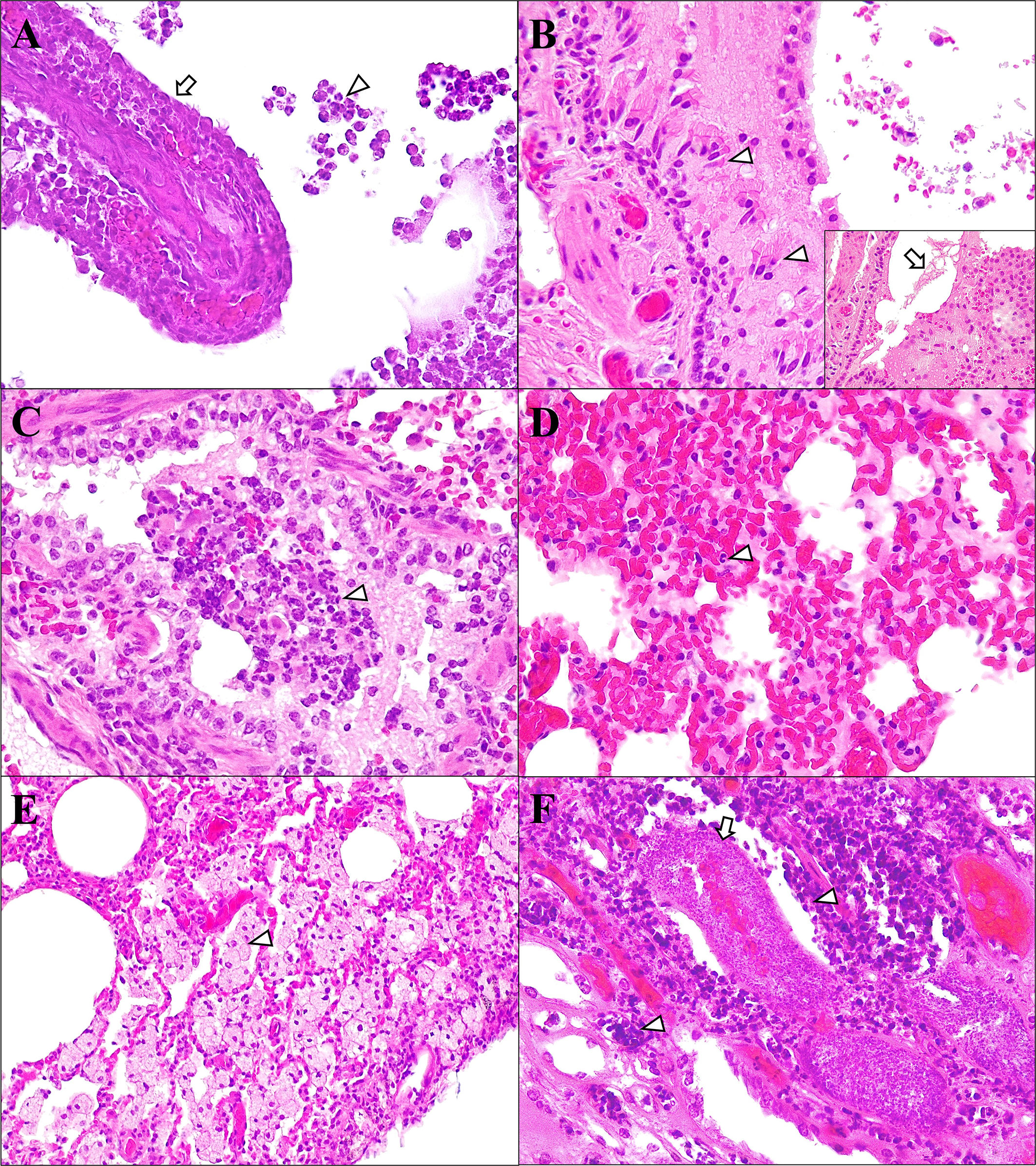

Supplement: SUPPLEMENTARY FIGURE S2 — Occasional histopathological findings observed in nasal turbinates (A); lung (B–E) and kidney at 7 dpi (G). (A) Severe erosive and limphoplasmocytic rhinits with removal of the cilia (arrow), and severe luminal neutrophil infiltration (arrowhead). (B) Bronchiolar epithelium with hyperplasia and epithelial detachment and pycnosis; the lumen was partially obstructed by an amorphous eosinophilic material, observing a slight deposit of erythrocytes, cellular debris and fibrin (arrow). (C) Moderate suppurative infiltration in the bronchiolar lumen (arrowhead); severe bronchus epithelial hyperplasia and detachment. Inset: perivascular lymphoplasmocytic cuffings. (D) Mild interstitial pneumonia with scarce number of neutrophils and moderate congestion. (E) Moderate intra-alveolar infiltrate of foamy macrophages (arrowhead). (F) Acute tubular necrosis and multifocal suppurative pyelonephritis (arrowhead), with the presence of bacterial colonies in the lumen of the medullary tubules (arrow). [file Image_2.TIF]

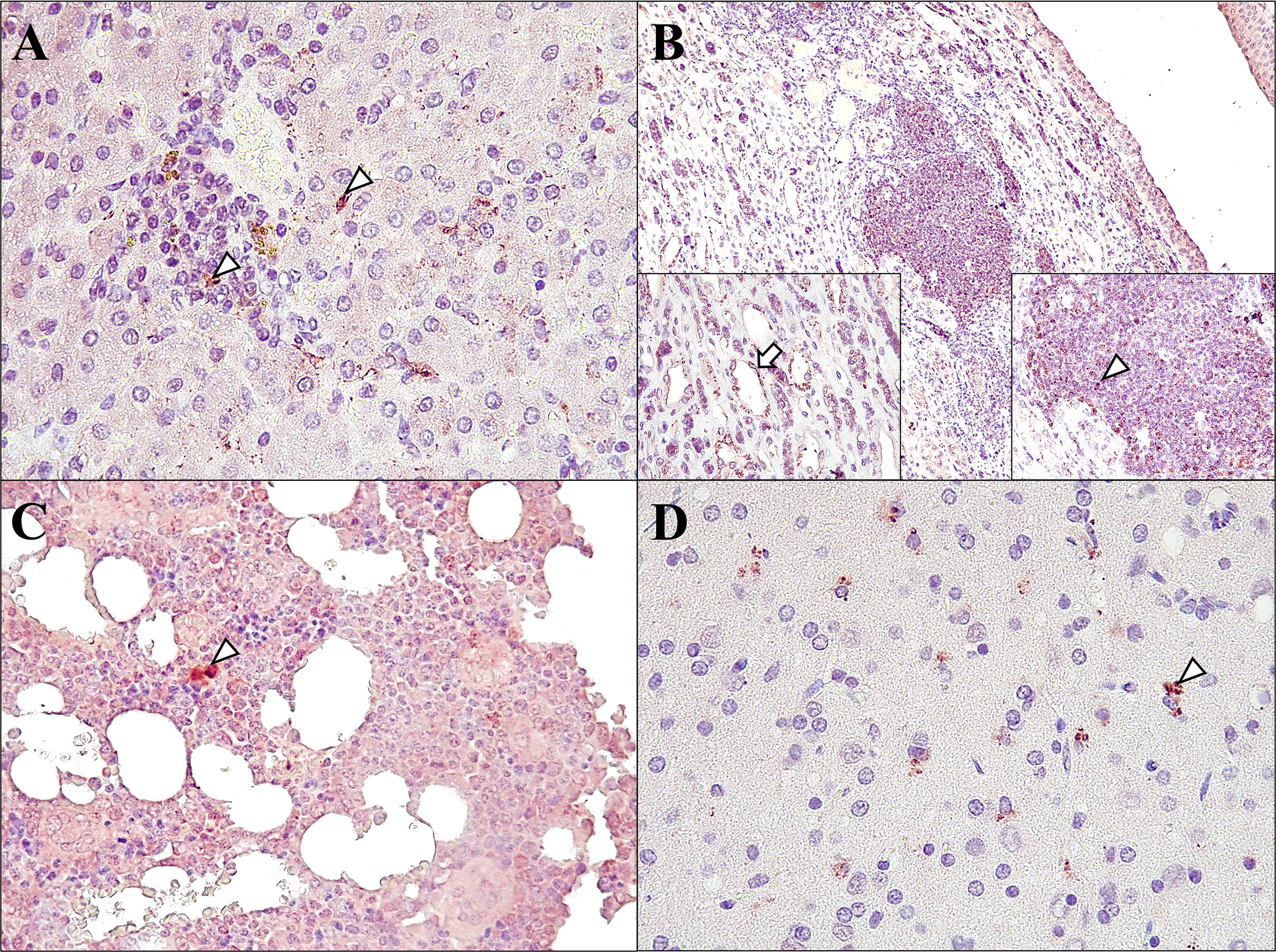

Supplement: SUPPLEMENTARY FIGURE S3 — SARS-CoV-2 distribution and cellular localization. Occasional immunohistochemical findings observed in liver (A), kidney (B), bone marrow (C) and brain (D) at 7 dpi. (A) Mild immunoexpression in the hepatocytes adjacent to periportal inflammation. (B) Intense immunolabeling adjacent to focal renal pelvis inflammation. Inset (left): immunolabeling in the basement membrane of renal tubules. Inset (right): immunolabeling in the cytoplasm of the inflammatory cells. (C) Mild immunoexpression in the cytoplasm of hematopoietic cells. (D) Mild immunoexpression in the cytoplasm of neurons and glial cells, mainly in the microglia (arrowhead). [file Image_3.TIF]

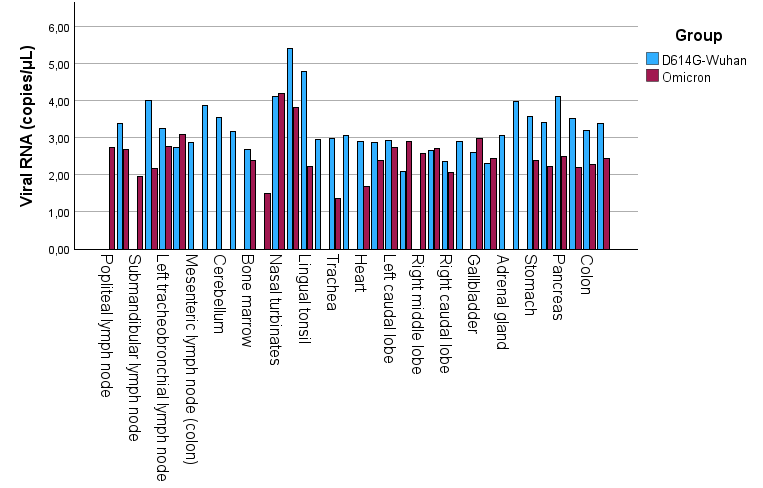

Supplement: SUPPLEMENTARY FIGURE S4 — Comparison of viral loads (copies per microliter) in positive tissues from ferrets infected with D614G-Wuhan (blue bars) and Omicron BA.5 (red bars) variants of SARS-CoV-2. [file Image_4.TIF]
