# Supplementary material for: Comparative SARS-CoV-2 Omicron BA.5 variant and D614G-Wuhan strain infections in ferrets: insights into attenuation and disease progression during subclinical to mild COVID-19
Source: Front Vet Sci. 2024 Aug 15;11:1435464. doi: 10.3389/fvets.2024.1435464 (PMC11358085; doi:10.3389/fvets.2024.1435464)
Supplement: SUPPLEMENTARY TABLE S3 — Histopathological scoring in tissues of SARS-CoV-2 infected ferrets. [file Table_3.DOCX]

**Supplementary Table 3.** Histopathological scoring in tissues of SARS-CoV-2 infected ferrets.

| **ORGAN** | **Histopathological finding*** | **WUHAN** | | | | | | **OMICRON** | | | | | |
| --- | --- | --- | --- | --- | --- | --- | --- | --- | --- | --- | --- | --- | --- |
|  |  | **7 DPI** | | **14 DPI** | | **21 DPI** | | **7 DPI** | | **14 DPI** | | **21 DPI** | |
|  |  | **H1** | **H2** | **H3** | **H4** | **H5** | **H6** | **H7** | **H8** | **H9** | **H10** | **H11** | **H12** |
| **Nasal turbinates** | Mononuclear/neutrophilic interstitial rhinitis | 3 | 1 | 1 | 2 | 0 | 0 | 0 | 0 | 1 | 1 | 0 | 0 |
|  | Segmental degeneration/necrosis of respiratory epithelium | 3 | 1 | 1 | 1 | 0 | 0 | 0 | 0 | 0 | 0 | 0 | 0 |
|  | Erythrocytes in nasal passages | 0 | 0 | 0 | 0 | 0 | 0 | 0 | 0 | 0 | 0 | 0 | 0 |
|  | Luminal cellular debris/degenerate neutrophils | 3 | 1 | 0 | 0 | 0 | 0 | 0 | 0 | 2 | 0 | 0 | 0 |
| **Trachea** | Mononuclear/neutrophilic tracheitis | 1 | 1 | 1 | 0 | 0 | 0 | 1 | 0 | 1 | 0 | 0 | 0 |
| **Lung** | Mononuclear/neutrophilic bronchointerstitial pneumonia | 2 | 2 | 2 | 3 | 2 | 2 | 0 | 2 | 2 | 1 | 2 | 1 |
|  | Interstitial edema | 2 | 2 | 2 | 2 | 2 | 2 | 0 | 2 | 1 | 0 | 1 | 0 |
|  | Alveolar edema | 2 | 3 | 3 | 2 | 3 | 2 | 2 | 2 | 2 | 2 | 1 | 2 |
|  | Septal thickening | 2 | 1 | 3 | 3 | 1 | 2 | 0 | 2 | 2 | 1 | 1 | 1 |
|  | Alveolar wall necrosis | 1 | 1 | 1 | 1 | 2 | 2 | 1 | 0 | 0 | 0 | 0 | 0 |
|  | Bronchial epithelial cell necrosis | 2 | 2 | 3 | 2 | 2 | 2 | 2 | 0 | 2 | 2 | 0 | 1 |
|  | Type II pneumocyte hyperplasia | 2 | 1 | 2 | 2 | 1 | 2 | 0 | 1 | 1 | 1 | 1 | 1 |
|  | Syncytial-like cells | 1 | 0 | 1 | 1 | 0 | 1 | 0 | 0 | 0 | 0 | 0 | 0 |
|  | Lymphocytic perivascular cuffings | 2 | 2 | 3 | 2 | 1 | 2 | 1 | 2 | 2 | 0 | 0 | 0 |
|  | Vasculitis | 0 | 0 | 0 | 0 | 0 | 0 | 0 | 0 | 0 | 0 | 0 | 0 |
|  | Vascular thrombosis | 0 | 0 | 0 | 0 | 0 | 0 | 0 | 0 | 0 | 0 | 0 | 0 |
|  | Alveolar/interstitial/perivascular heamorrhage | 1 | 1 | 0 | 1 | 0 | 0 | 0 | 0 | 0 | 0 | 0 | 0 |
| **Liver** | Mononuclear/neutrophilic hepatitis | 2 | 1 | 1 | 3 | 2 | 3 | 1 | 1 | 2 | 3 | 0 | 2 |
|  | Hepatocellular necrosis | 1 | 0 | 2 | 1 | 2 | 2 | 0 | 0 | 0 | 2 | 2 | 1 |
| **Kidney** | Mononuclear/neutrophilic interstitial nephritis | 2 | 0 | 0 | 0 | 0 | 0 | 0 | 0 | 1 | 0 | 0 | 0 |
| **Spleen** | Lymphocytolisis | 0 | 0 | 0 | 0 | 0 | 0 | 0 | 0 | 0 | 0 | 0 | 0 |
|  | Lymphoid depletion | 0 | 0 | 0 | 0 | 0 | 0 | 0 | 0 | 0 | 0 | 0 | 0 |
|  | Follicular hyperplasia | 2 | 2 | 2 | 2 | 2 | 2 | 2 | 2 | 2 | 2 | 2 | 2 |
|  | Mononuclear/neutrophilic splenitis | 0 | 0 | 0 | 0 | 0 | 0 | 0 | 0 | 0 | 0 | 0 | 0 |
|  | Hyperemia | 3 | 2 | 3 | 2 | 3 | 3 | 3 | 3 | 3 | 3 | 3 | 3 |
|  | Megacaryocytes | 0 | 0 | 0 | 0 | 0 | 0 | 0 | 0 | 0 | 0 | 0 | 0 |
| **Lymph nodes** | Lymphocytolisis | 0 | 0 | 0 | 0 | 0 | 0 | 0 | 0 | 0 | 0 | 0 | 0 |
|  | Lymphoid depletion | 0 | 0 | 0 | 0 | 0 | 0 | 0 | 0 | 0 | 0 | 0 | 0 |
|  | Follicular hyperplasia | 3 | 2 | 2 | 2 | 1 | 2 | 2 | 2 | 1 | 2 | 1 | 2 |
|  | Mononuclear/neutrophilic lymphadenitis | 1 | 1 | 0 | 0 | 0 | 0 | 0 | 1 | 0 | 0 | 0 | 1 |
| **Brain** | Non-suppurative meningoencephalitis | 1 | 1 | 1 | 1 | 1 | 1 | 0 | 0 | 1 | 1 | 1 | 1 |
|  | Lymphocytes in choroid plexus/subependymal space | 1 | 0 | 2 | 1 | 1 | 2 | 0 | 0 | 0 | 1 | 1 | 2 |
|  | Perivascular lymphocytic cuffings | 0 | 0 | 1 | 2 | 1 | 1 | 0 | 0 | 1 | 0 | 1 | 1 |
|  | Vasculitis | 0 | 0 | 0 | 0 | 0 | 0 | 0 | 0 | 0 | 0 | 0 | 0 |
|  | Perivascular haemorrhage | 0 | 0 | 0 | 0 | 0 | 0 | 0 | 0 | 0 | 0 | 0 | 0 |
|  | Thrombosis | 0 | 0 | 0 | 0 | 0 | 0 | 0 | 0 | 0 | 0 | 0 | 0 |
|  | Neuropil spongiosis (cytoplasmic ballooning). | 0 | 0 | 1 | 2 | 0 | 0 | 0 | 0 | 0 | 0 | 0 | 0 |
|  | White matter tract myelin sheath vacuolation | 0 | 0 | 0 | 0 | 0 | 0 | 0 | 0 | 0 | 0 | 0 | 0 |
|  | Microglial activation (neuronophagia) | 2 | 1 | 2 | 1 | 2 | 1 | 1 | 1 | 1 | 1 | 1 | 1 |
|  | Oligodendrocyte activation (satellitosis) | 2 | 1 | 3 | 2 | 2 | 2 | 2 | 1 | 2 | 1 | 1 | 1 |
|  | Astroglial activation (astrogliosis) | 1 | 1 | 2 | 2 | 2 | 2 | 2 | 2 | 2 | 2 | 1 | 1 |

*Histopathological score: 0 (negative); 1 (mild); 2 (moderate); 3 (severe).
