# Supplementary material for: Comparative SARS-CoV-2 Omicron BA.5 variant and D614G-Wuhan strain infections in ferrets: insights into attenuation and disease progression during subclinical to mild COVID-19
Source: Front Vet Sci. 2024 Aug 15;11:1435464. doi: 10.3389/fvets.2024.1435464 (PMC11358085; doi:10.3389/fvets.2024.1435464)
Supplement: SUPPLEMENTARY TABLE S4 — Immunohistochemical scoring to determine the presence of virus in SARS-CoV-2 infected ferret tissues. [file Table_4.DOCX]

**Supplementary Table 4.** Immunohistochemical scoring to determine the presence of virus in SARS-CoV-2 infected ferret tissues.

| **ORGAN** | **Immunoexpression***  **(SARS-COV-2)** | **WUHAN** | | | | | | **OMICRON** | | | | | |
| --- | --- | --- | --- | --- | --- | --- | --- | --- | --- | --- | --- | --- | --- |
|  |  | **7 DPI** | | **14 DPI** | | **21 DPI** | | **7 DPI** | | **14 DPI** | | **21 DPI** | |
|  |  | **H1** | **H2** | **H3** | **H4** | **H5** | **H6** | **H7** | **H8** | **H9** | **H10** | **H11** | **H12** |
| **Nasal turbinates** | Olfatory epithelium | 2 | 2 | 0 | 0 | 1 | 0 | 1 | 2 | 0 | 0 | 0 | 0 |
|  | Exudate | 3 | 0 | 0 | 0 | 0 | 0 | 0 | 0 | 0 | 0 | 0 | 0 |
| **Trachea** | Respiratory epithelium | 1 | 1 | 0 | 0 | 0 | 0 | 0 | 0 | 0 | 0 | 0 | 0 |
| **Lung** | Bronchial/bronchiolar epithelial cells | 2 | 2 | 0 | 0 | 0 | 0 | 2 | 2 | 0 | 0 | 0 | 0 |
|  | Intralveolar cells (macrophages/pneumocytes) | 3 | 1 | 0 | 0 | 0 | 0 | 2 | 1 | 0 | 0 | 0 | 0 |
| **Liver** | Vascular and sinusoidal spaces | 1 | 1 | 0 | 0 | 0 | 0 | 0 | 0 | 0 | 0 | 0 | 0 |
|  | Kupffer cells | 1 | 1 | 0 | 0 | 0 | 0 | 0 | 0 | 0 | 0 | 0 | 0 |
|  | Hepatocytes | 2 | 1 | 0 | 0 | 0 | 0 | 0 | 0 | 0 | 0 | 0 | 0 |
|  | Inflammatory cells | 1 | 1 | 0 | 0 | 0 | 0 | 0 | 0 | 0 | 0 | 0 | 0 |
| **Kidney** | Tubular cells | 3 | 0 | 0 | 0 | 0 | 0 | 0 | 0 | 0 | 0 | 0 | 0 |
|  | Inflammatory cells | 2 | 0 | 0 | 0 | 0 | 0 | 0 | 0 | 0 | 0 | 0 | 0 |
| **Spleen** | Macrophages | 1 | 2 | 0 | 0 | 0 | 0 | 0 | 0 | 0 | 0 | 0 | 0 |
| **Lymph nodes** | Macrophages | 1 | 1 | 0 | 0 | 0 | 0 | 1 | 1 | 0 | 0 | 0 | 0 |
| **Bone marrow** | Macrophages | 1 | 0 | 0 | 0 | 0 | 0 | 1 | 1 | 0 | 0 | 0 | 0 |
| **Brain** | Neurons | 2 | 1 | 0 | 0 | 0 | 0 | 0 | 0 | 0 | 0 | 0 | 0 |
|  | Glial cells | 1 | 0 | 0 | 0 | 0 | 0 | 0 | 0 | 0 | 0 | 0 | 0 |

*Immunohistochemical score (SARS-CoV-2): 0 (negative); 1 (mild); 2 (moderate); 3 (severe).
